# An in silico model of retinal cholesterol dynamics (RCD model): insights into the pathophysiology of dry AMD[S]

**DOI:** 10.1194/jlr.M074088

**Published:** 2017-04-25

**Authors:** Seyedeh Maryam Zekavat, James Lu, Cyrille Maugeais, Norman A. Mazer

**Affiliations:** Biological Engineering,* Massachusetts Institute of Technology, Cambridge, MA; and; Departments of Clinical Pharmacology† and Neuroscience, Ophthalmology, and; Rare Diseases,§ Roche Innovation Center Basel, Basel, Switzerland

**Keywords:** cell/tissue, eye/retina, high density lipoprotein/metabolism, low density lipoprotein/metabolism, lipoproteins/kinetics, lipoproteins/metabolism, macrophages/monocytes, membranes, transport, age-related macular degeneration

## Abstract

We developed an in silico mathematical model of retinal cholesterol (Ch) dynamics (RCD) to quantify the physiological rate of Ch turnover in the rod outer segment (ROS), the lipoprotein transport mechanisms by which Ch enters and leaves the outer retina, and the rates of drusen growth and macrophage-mediated clearance in dry age-related macular degeneration. Based on existing experimental data and mechanistic hypotheses, we estimated the Ch turnover rate in the ROS to be 1–6 pg/mm^2^/min, dependent on the rate of Ch recycling in the outer retina, and found comparable rates for LDL receptor-mediated endocytosis of Ch by the retinal pigment epithelium (RPE), ABCA1-mediated Ch transport from the RPE to the outer retina, ABCA1-mediated Ch efflux from the RPE to the choroid, and the secretion of 70 nm ApoB-Ch particles from the RPE. The drusen growth rate is predicted to increase from 0.7 to 4.2 μm/year in proportion to the flux of ApoB-Ch particles. The rapid regression of drusen may be explained by macrophage-mediated clearance if the macrophage density reaches ∼3,500 cells/mm^2^. The RCD model quantifies retinal Ch dynamics and suggests that retinal Ch turnover and recycling, ApoB-Ch particle efflux, and macrophage-mediated clearance may explain the dynamics of drusen growth and regression.

Cholesterol (Ch) molecules play both physiological and pathological roles in the outer retina. In the physiological context, Ch is an essential component of the rod outer segment (ROS) discs, stacked membrane structures containing rhodopsin, which form continuously in the rod inner segment (RIS) and migrate over ∼11 days to the retinal pigment epithelium (RPE) where they are phagocytosed (1, 2). In the pathological context, esterified and unesterified Ch together with phospholipids (PLs) constitute more than 40% of the volume of “drusen deposits,” the hallmark of dry age-related macular degeneration (AMD) (3–5). Drusen slowly accumulate in Bruch’s membrane (BrM) over decades (3, 6) and, in some cases, have been observed to regress rapidly prior to the development of advanced forms of AMD, geographic atrophy, and neovascular AMD (7–10). The source of drusen Ch is thought to be the RPE (11, 12), which under physiological conditions eliminates the phagocytosed Ch via ABCA1-mediated efflux to lipid-poor ApoA-I (13, 14), but under pathological conditions secretes esterified and unesterified Ch in large Apo-B-containing particles that become entrapped in the BrM as basal linear deposits and drusen (3, 12, 15–17). The mechanism of drusen regression has not been fully identified, but may involve the infiltration of activated macrophages, which ingest the drusen Ch and eliminate it via an ABCA1-mediated process (10, 18, 19).

Based on existing experimental data (2, 11, 20–23) and mechanistic hypotheses (3, 12–17), we have developed an in silico model of retinal Ch dynamics (RCD) in an effort to quantitatively understand both the physiological and pathophysiological processes associated with Ch dynamics in the outer retina and their interrelationships. In contrast to earlier in silico models of retinal physiology and disease states (24–26), the RCD model was designed to address the following questions: *1*) What is the physiological rate of Ch turnover in the outer retina and what mechanisms govern it? *2*) How is Ch provided to the outer retina (via delivery by lipoproteins versus local synthesis)? *3*) How does Ch leave the outer retina? *4*) What determines the slow rate of drusen growth in dry AMD? *5*) What processes are responsible for the rapid rates of drusen regression observed in some patients?

The RCD model consists of three modules that are illustrated schematically in **Fig. 1**. Module 1 represents the mechanisms of Ch turnover in the ROS, which include: formation of Ch-rich discs in the RIS, Ch removal from the discs as they migrate down the ROS, and phagocytosis of Ch-depleted discs by the RPE. We hypothesize that the Ch removed from discs enters a “recycling compartment” from which it may either be recycled to the RIS for new disc formation or enter the RPE cells for elimination. Although the mechanisms responsible for Ch removal and recycling are not known, Fliesler and Bretillon (13) have suggested that ApoB, ApoA-I, and the cholesteryl ester transport protein (CETP) may be involved. We speculate that the recycling compartment itself may be located either in the inter-photoreceptor matrix or the ROS plasma membrane, which encases the discs. Module 2 represents the mechanisms of Ch input from the choroidal capillaries (CCs) to the outer retina. Ch delivery from the CC is modeled as a three step process that incorporates: *1*) transcytosis of LDL across the CC endothelium, *2*) LDL receptor-mediated influx into RPE cells, and *3*) ABCA1-mediated transport of Ch to the rods. Finally, module 3 represents Ch efflux from the basal RPE to the BrM via two pathways: *1*) the “physiological” mode of ABCA1-mediated transport of intracellular Ch to Ch-poor ApoA-I molecules that are cleared by the CCs; and *2*) the “pathological” mode in which large ApoB-Ch particles are effluxed into the BrM where they are entrapped as basal linear deposits and drusen. Module 3 also includes the presumed role of macrophages that infiltrate drusen, ingest Ch, and eliminate it via the ABCA1 pathway.

**Fig. 1.**
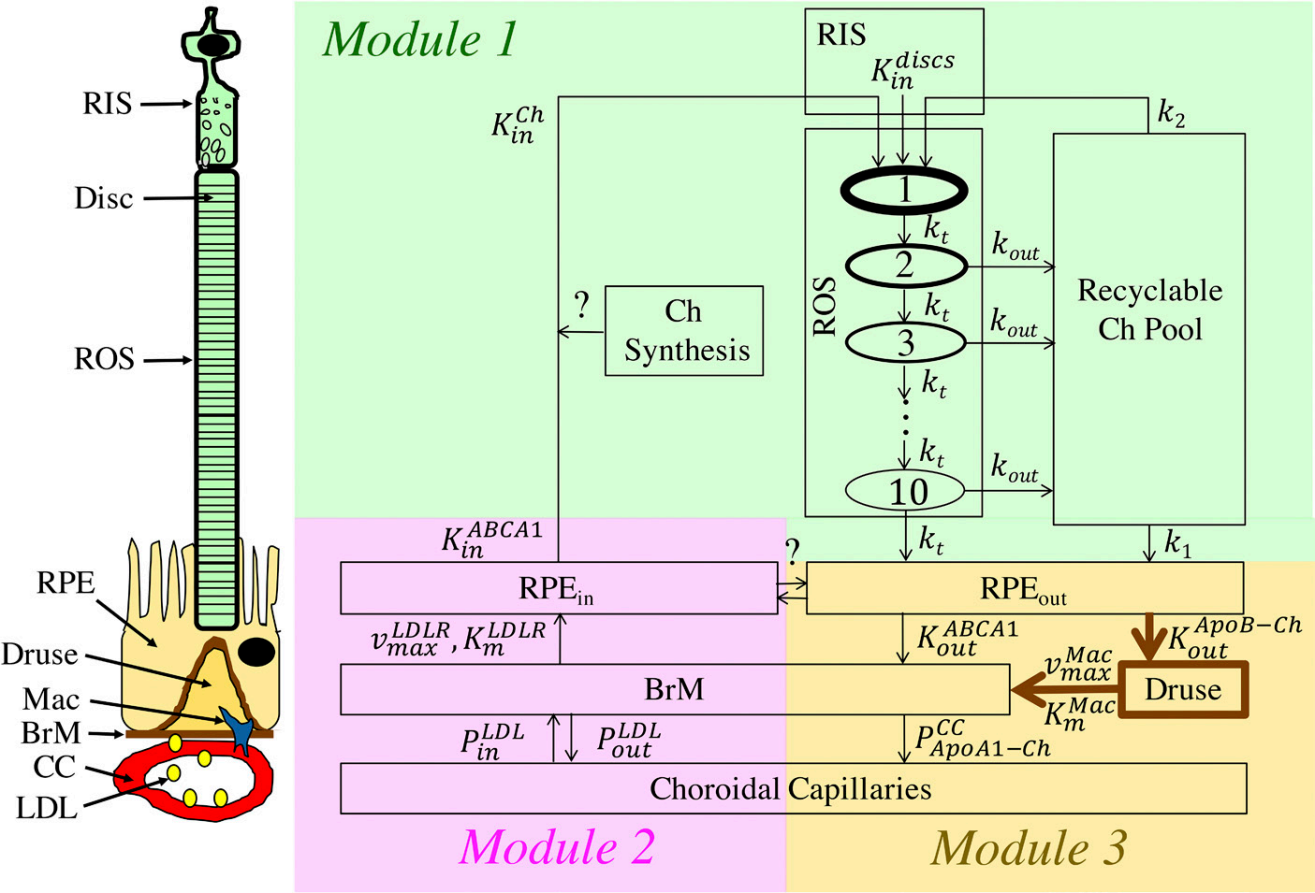
Modular structure of the RCD model. The cartoon on the left depicts (from bottom to top): a choroidal capillary (CC) with LDL particles, Bruch’s membrane (BrM), macrophage (Mac), Druse, retinal pigment epithelial cell (RPE), rod outer segment (ROS), and rod inner segment (RIS) where disc membrane components are synthesized. Module 1 (green-shaded area) portrays Ch turnover in the outer retina. Ch enters the RIS with flux $K_{in}^{Ch}$, which incorporates both Ch delivery from the CC and the possibility of Ch synthesis. In the RIS, Ch is integrated into newly synthesized discs which enter the ROS with rate $K_{in}^{discs}$. The ROS is divided into 10 transit-chain compartments (27), each containing 100 discs. During their ∼11 day transit through the ROS (with rate constant *k_t_* between each compartment), the Ch content of the discs decreases by a first-order process with rate constant *k_out_*. Discs in the last transit compartment are phagocytosed by the RPE by a first-order process with rate constant *k_t_*. We hypothesize that Ch leaving the discs enters a “Recyclable Ch Pool” from which it may either be recycled back to the RIS and used toward the formation of new Ch-containing discs (with first-order rate constant *k*_2_), or taken up by RPE cells (with first-order rate constant *k*_1_). Module 2 (pink-shaded area) portrays the delivery of Ch from the CC to the RPE and thereafter to the outer retina via a three-step process: *1*) bidirectional transcytosis of LDL particles across the CC endothelium and into the BrM (modeled by effective permeability coefficients, $P_{in}^{LDL}$ and $P_{out}^{LDL}$), *2*) LDLR-mediated uptake of LDL particles into RPE cells (modeled by Michaelis-Menten kinetics (29, 43, 44) with parameters $v_{max}^{LDLR}$, $\;K_{m}^{LDLR}$), and *3*) ABCA1-mediated transport of Ch apically from RPE cells to lipid-poor ApoA-I and thereby into the rod layer (13) (with flux $K_{in}^{ABCA1}$). Module 3 (yellow-shaded area) portrays Ch efflux from the basal RPE to the BrM via two pathways: *1*) the physiological mode of ABCA1-mediated transport of intracellular Ch to Ch-poor ApoA-I molecules (with flux $K_{out}^{ABCA1}$) that are cleared by the choroidal circulation; and *2*) the pathological mode in which large ApoB-Ch particles are effluxed into the BrM (with flux $K_{out}^{ApoB\text{-}Ch}$) where they are entrapped as basal linear deposits and drusen. Macrophages are hypothesized to clear druse Ch via ABCA1-mediated efflux to ApoA-I-Ch particles (modeled using Michaelis-Menten kinetics with parameters $\;v_{max}^{Mac}$ and $\;K_{m}^{Mac}$). The ApoA-I-Ch particles produced via ABCA1-mediated transport by the basal RPE or macrophages are presumed to be small enough to permeate into the CC (with permeability coefficient $P_{ApoAI\text{-}Ch}^{CC}$). With the exception of the linkage between module 1 (Ch turnover rate) and module 3 (drusen growth rate), the predicted fluxes in each module do not explicitly interact with each other.

Based on the analysis of the RCD model and its modules, our principle findings are: *1*) the Ch turnover rate in the outer retina decreases from ∼6 to ∼1 pg/mm^2^/min as the extent of Ch recycling varies from 0% to 100%; *2*) LDL particle uptake by the RPE can provide the necessary amount of Ch for Ch turnover and thus, Ch synthesis is not needed in the outer retina; *3*) the ApoA-I and ApoB pathways can both efflux Ch at rates that are comparable to the estimated range of Ch turnover rates; *4*) if ApoB particles alone efflux Ch, the thickness of the deposited Ch layer will increase at rates of 0.7–4.2 μm/year (corresponding to the 6-fold range in Ch turnover rate) and, thereby, account for the slow appearance of drusen over decades; and *5*) macrophage densities of 3,500 cells/mm^2^ are required to explain the rapid rate of drusen regression seen by optical coherence tomography in some patients with dry AMD (9).

As a caveat to these findings, it should be noted that while the estimates of Ch turnover and several other parameters were derived from retinal data in humans and other species, the other RCD calculations were derived indirectly from data on hepatic lipoprotein metabolism and scaled by geometric considerations. Further experimental data are therefore needed to validate and/or refine the hypotheses and results presented here.

## METHODS

### Compartmental modeling of RCD pathways

Here we describe the biological mechanisms that are assumed to operate within the three modules of the RCD model (shown in Fig. 1) and the computational approaches used to describe them. Details on the calculations themselves are provided in the corresponding sections of the supplemental material.

### Module 1: Ch turnover in the ROS

**Fig. 2.**
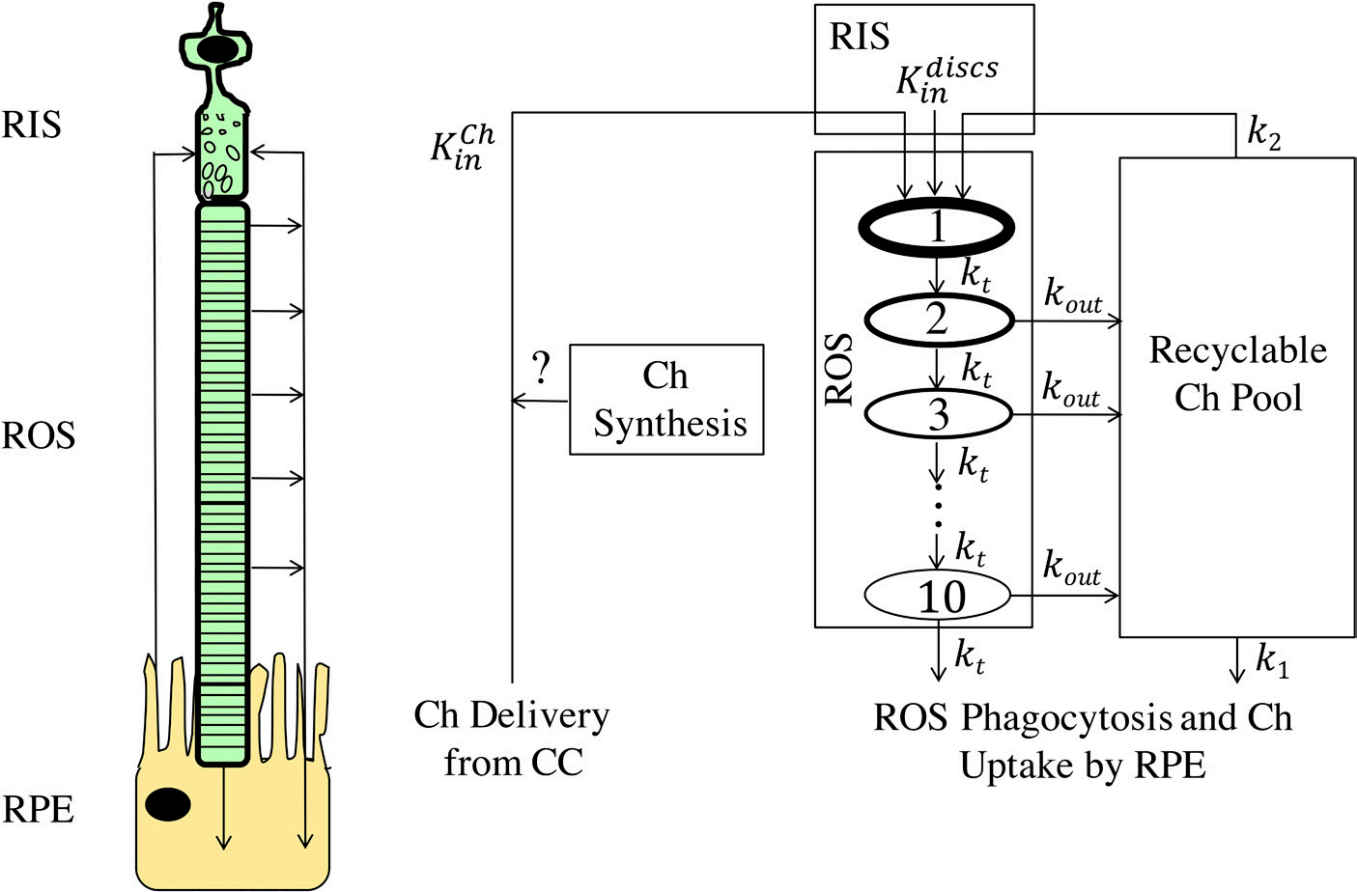
Compartmental structure of module 1. The cartoon on the left depicts Ch movement between RPE RIS, and ROS. Compartments and arrows on the right correspond to hypothesized pools and mechanisms that affect Ch turnover in the outer retina. Ch enters the RIS with flux $K_{in}^{Ch}$, which incorporates both Ch delivery from the CC and the possibility of Ch synthesis. In the RIS, Ch is integrated into newly synthesized discs, which enter the ROS with rate $K_{in}^{discs}$. The ROS is divided into 10 transit-chain compartments (27), each containing 100 discs. During their ∼11 day transit through the ROS (with rate constant *k_t_* between each transit-chain compartment), the Ch content of the discs decreases by a first-order process with rate constant *k_out_*. Discs in the last transit compartment are phagocytosed by the RPE by a first-order process with rate constant *k_t_*. We hypothesize that Ch leaving the discs enters a “Recyclable Ch Pool” from which it may either be recycled back to the RIS and used toward the formation of new Ch-containing discs (with first-order rate constant *k*_2_), or taken up by RPE cells (with first-order rate constant *k*_1_). See supplemental material S1 for further details.

As shown in **Fig. 2** and described in supplemental material S1, module 1 of the RCD model used a transit-chain model (27) to describe the formation and movement of Ch-containing discs through the ROS and to compute the associated fluxes of Ch into and out of discs. Ch enters the RIS with a flux $K_{in}^{Ch}$, which incorporates both Ch delivery from the CC and the possibility of Ch synthesis. In the RIS, Ch is integrated into newly synthesized discs, which enter the ROS with rate $K_{in}^{discs}$. The ROS is divided into 10 transit-chain compartments, each containing 100 discs. The discs pass through the ROS with a mean transit time of ∼11 days (2) and are phagocytosed by the RPE from the last compartment by a first-order process with rate constant *k_t_*. During its transit, the Ch content of the discs decreases by approximately 6-fold (25, 26). The first-order rate constant *k_out_* describes the rate of Ch efflux from the discs in each transit-chain compartment. Although it is not known how Ch is effluxed from ROS discs, we hypothesized that Ch enters a “recyclable pool” from which it may either be recycled back into the RIS and used toward the formation of new Ch-containing discs (with first-order rate constant *k*_2_), or taken up by RPE cells (with first-order rate constant *k*_1_). We speculate that the recyclable pool may be located within the inter-photoreceptor matrix or the ROS plasma membrane, which encases the discs. The fractional recycling of Ch in module 1 was quantified as: *f_recycled_* = *k*_2_/(*k*_1_ + *k*_2_). At steady-state, the influx rate of Ch into the RIS, $K_{in}^{Ch}$, is equal to the combined rates at which Ch is phagocytosed from the last ROS disc compartment and taken up from the recyclable pool by the RPE, and corresponds to the Ch turnover rate in the ROS.

### Module 2: delivery of Ch from CC to RPE to outer retina

**Fig. 3.**
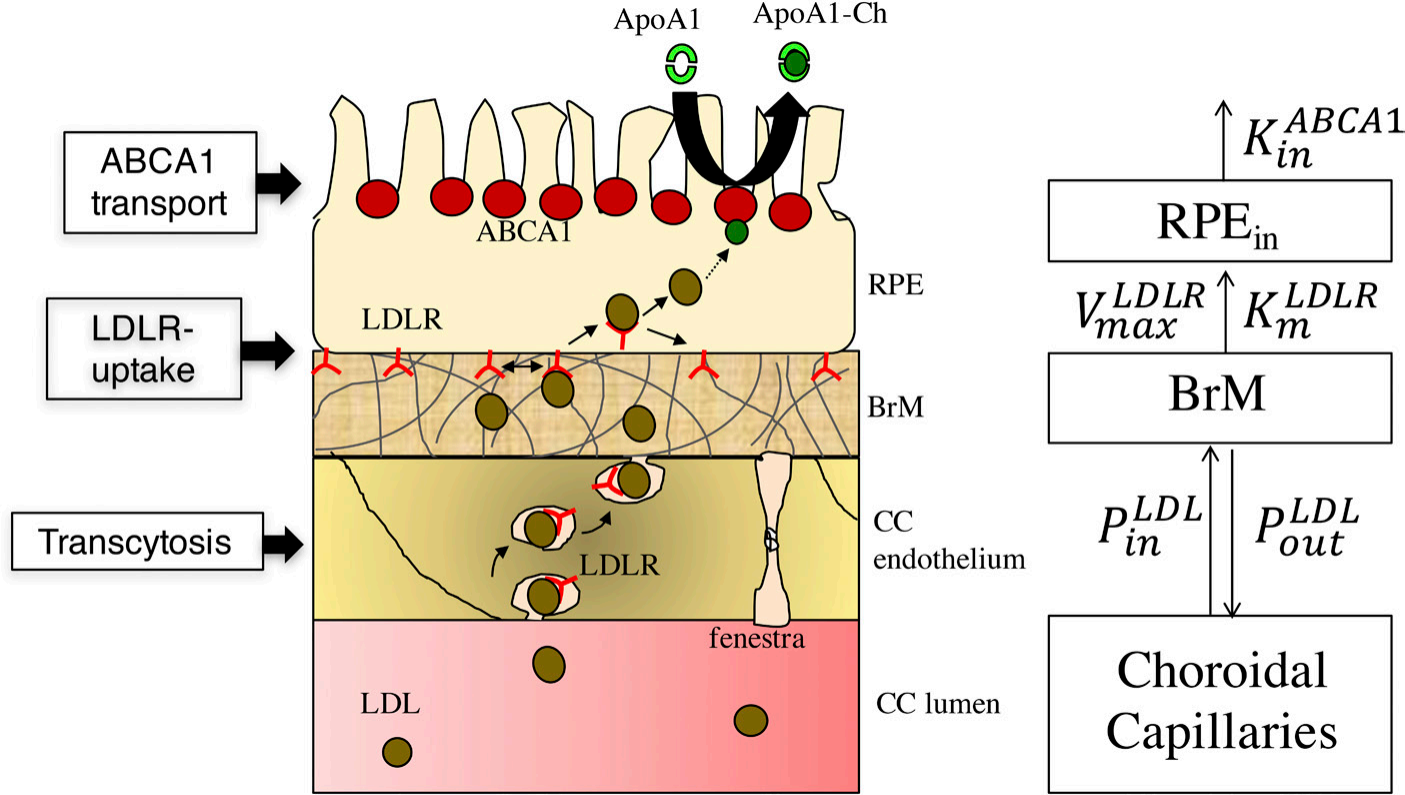
Compartmental structure of module 2. The cartoon on left depicts (from bottom to top) the three steps of Ch delivery to the outer retina: transcytosis across the CC endothelium, LDLR-mediated uptake into the RPE, and ABCA1-mediated transport from the apical surface of the RPE to the outer retina. Compartments and arrows on right show: *1*) modeling of transcytosis between CC and BrM using effective permeability coefficients, $P_{in}^{LDL}$ and $P_{out}^{LDL}$; *2*) receptor-mediated uptake of LDL particles by the RPE by Michaelis-Menten kinetics of LDLR (29, 43, 44) with parameters $v_{max}^{LDLR}$, $\;K_{m}^{LDLR}$; and *3*) ABCA1-mediated transport from the RPE to lipid-poor ApoA-I particles with flux $K_{in}^{ABCA1}$. See supplemental material S2–S4 for further details.

Several lines of evidence indicate that circulating LDL is a major source of Ch for the outer retina (28–35). In module 3 (**Fig. 3**), the delivery of Ch into the outer retina is modeled via a three-step process: *1*) transcytosis of LDL-Ch across the CC endothelium and into the BrM; *2*) LDL receptor (LDLR)-mediated influx of LDL-Ch into RPE cells; and *3*) ABCA1-transport of Ch apically from RPE cells to lipid-poor ApoA-I and, thereby, into the rod layer (13). Because LDL particles have an average diameter of 21 nm (36, 37), it is unlikely that they can pass through the diaphragmed fenestra of the CC, which have pore sizes of ∼6–12 nm (38). We therefore assumed in step 1 that LDL passes across the CC endothelium by transcytosis (receptor-mediated vesicular transport), as suggested for other capillary beds (39–41). As detailed in supplemental material S2, we modeled transcytosis into and out of the BrM via effective permeability coefficients, $P_{in}^{LDL}$ and $P_{out}^{LDL}$, which were estimated from data on LDL transcytosis across the nonfenestrated capillaries of the blood-brain barrier (42). In step 2, we modeled the receptor-mediated uptake of LDL particles by the RPE assuming Michaelis-Menten kinetics of LDLR (29, 43, 44), as detailed in supplemental material S3. Based on experimental data (45), we assumed in step 3 that Ch is delivered from the apical surface of the RPE to the outer retina via ABCA1-mediated transport to lipid-poor ApoA-I particles (with flux $K_{in}^{ABCA1}$). As detailed in supplemental material S4, we derived an estimate of the rate of ABCA1-mediated transport of Ch from the apical RPE surface, $K_{in}^{ABCA1}$, based on the rate of hepatic ABCA1-mediated transport via the reverse Ch transport pathway.

### Module 3: efflux of Ch from RPE to BrM to CC

**Fig. 4.**
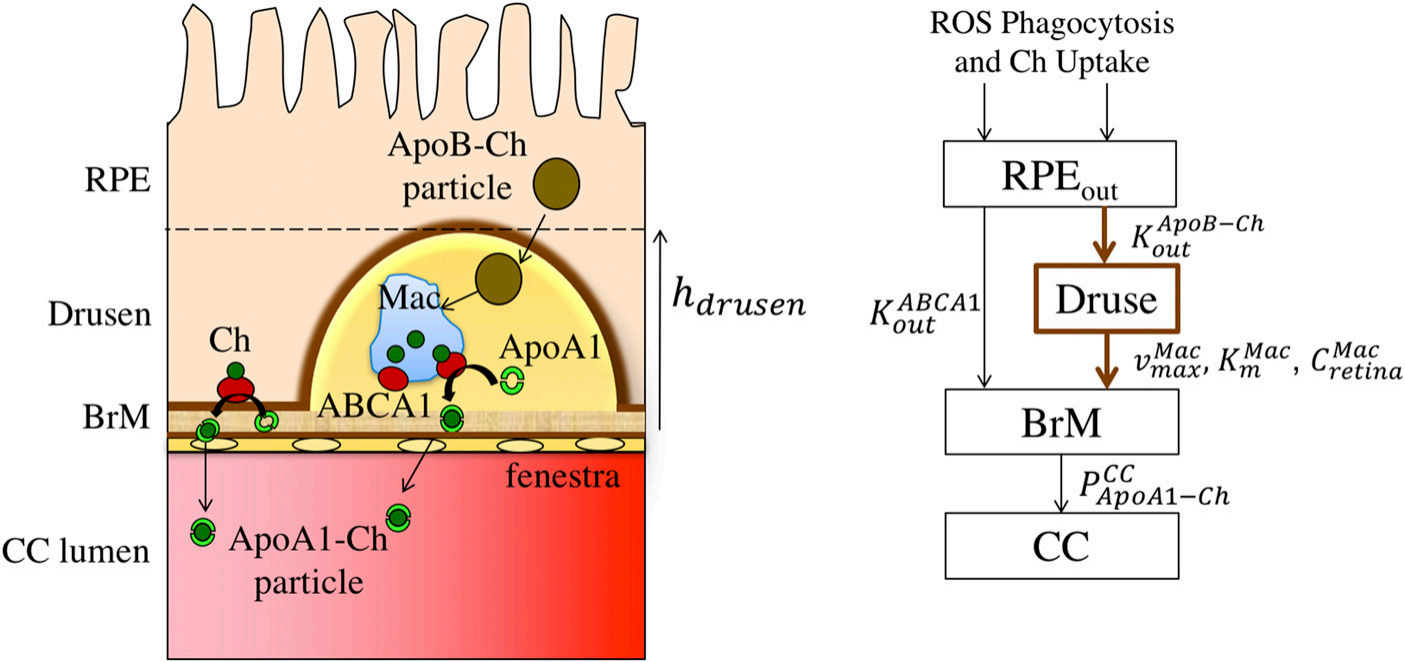
Compartmental structure of module 3. The cartoon on left depicts Ch efflux from the RPE via small ApoA-I-Ch particles (the physiological pathway) and large ApoB-Ch particles (the pathological pathway leading to drusen). The small ApoA-I-Ch particles are generated via the ABCA1 transporter, transit across the BrM, and enter the CC via endothelial cell fenestrations. The large ApoB-Ch particles are assumed to be generated by a process that involves the microsomal transport protein (MTP) and accumulate in the BrM as drusen. Macrophages can phagocytose the drusen Ch and efflux it via ABCA1 to small ApoA-I-Ch particles similar to those generated from the RPE. The compartments and arrows on the right portray the ABCA1-mediated formation of small ApoA-I-Ch particles from the RPE (with flux $\;K_{out}^{ABCA1}$), the formation of large ApoB-Ch particles (with flux $K_{out}^{ApoB\text{-}Ch}$), which accumulate in drusen and the macrophage-mediated drusen clearance rate modeled using Michaelis-Menten-like kinetics (with parameters $\;v_{max}^{Mac}$, $\;K_{m}^{Mac}$, and the retinal macrophage concentration $C_{Retina}^{Mac}$). The permeability coefficient of the small ApoA-I-Ch particles across the CC is given by $\;P_{ApoA1\text{-}Ch}^{CC}$. See supplemental material S5–S9 for further details.

As shown in module 1 (Fig. 2), Ch enters the RPE by phagocytosis of ROS discs and uptake from the recyclable pool. In module 3 (**Fig. 4**) , two pathways of Ch efflux from the RPE to the BrM are represented: ABCA1-mediated Ch efflux from the basal surface of the RPE into ApoA-I-Ch particles (with flux $K_{out}^{ABCA1}$) and the secretion of large ApoB-Ch particles from the RPE (with flux $K_{out}^{ApoB\text{-}Ch}$), which accumulate in the BrM as drusen deposits (15–17). In the first pathway, $K_{out}^{ABCA1}$ was estimated from the rate at the apical surface $K_{in}^{ABCA1}$ based on experimental data of ABCA1 expression in the apical versus basal surfaces of the RPE (see supplemental material S5 for details). The resulting ApoA-I-Ch particles were small enough (6.3 nm in diameter) to permeate through the diaphragmed fenestra of the CC endothelium. As detailed in supplemental material S6, we estimated the permeability of the ApoA-I-Ch particles across the CC $\left(P_{ApoA\text{-}I\text{-}Ch}^{CC}\right)$ by combining the theory for permeation of particles through cylindrical pores (46–49) with experimental data on the density of diaphragmed fenestra in the CC (50–52), the number of pores within each fenestra (38), and the ratio of the ApoA-I-Ch particle size-to-pore size. The large ApoB-Ch particles secreted into the BrM (∼70 nm in diameter) are too large to pass through the diaphragmed fenestra and built up in the BrM over decades (53), forming basal linear deposits and drusen (15–17). As detailed in supplemental material S7, the capacity of RPE cells to efflux Ch via ApoB-containing particles was estimated using ApoB-Ch efflux from the liver, normalizing this rate to the secretory surface area of the hepatocyte, and adjusting for the differences in microsomal transport protein (MTP)-A and ApoB mRNA expression in RPE cells versus hepatocytes (54). The estimated rate of Ch deposition in the BrM is based on a number of key assumptions that are detailed in supplemental material S8. These assumptions imply that the drusen thickness (which combines the drusen and the basal linear deposit) will increase linearly in time and can be expressed as a drusen growth rate, $dh_{drusen}^{growth}/dt$, that is proportional to $K_{in}^{Ch}$. We note that the calculated Ch fluxes include the contributions of both unesterified and esterified Ch in the respective particles and in the deposited Ch.

Module 3 was completed by incorporating macrophage-­mediated clearance of Ch from the drusen deposits. As detailed in supplemental material S9, we assumed that the rate-limiting step in this process is via ABCA1-mediated efflux to lipid-poor ApoA-I and modeled this using Michaelis-Menten kinetics. The rate of drusen clearance $\left(dh_{drusen}^{Mac}/dt\right)$ depends on the maximum rate of ABCA1-mediated efflux per macrophage $\left(v_{max}^{Mac}\right)$, the macrophage density in the retina $\left(C_{retina}^{Mac}\right)$, estimated from the experimental data of Penfold, Killingsworth, and Sarks (55), the local concentration of lipid-poor ApoA-I, and the value of the Michaelis constant $K_{m}^{Mac}$. As noted above for the ABCA1-mediated efflux from the RPE, the small ApoA-I-Ch particles effluxed from macrophages may exit the BrM via the fenestrated endothelium of the CC with the same permeability $P_{ApoA1\text{-}Ch}^{CC}$.

### Additional assumptions in the integrated RCD model

As indicated in Fig. 1, we assumed that the RPE contains separate functional pools for the delivery of Ch to the retina (*RPE_in_*) and for the efflux of Ch to the BrM (*RPE_out_*), rather than a common pool in the RPE. This assumption is consistent with recent studies of hepatic Ch metabolism, which show that LDL particle intake is shunted through the hepatocyte for VLDL secretion and does not equilibrate with the regulatory pool that controls Ch synthesis and LDLR expression (56). We also note that the contribution of retinal Ch synthesis as a source for $K_{in}^{Ch}$ has not been conclusively determined in humans (13), but is assumed to be negligible in our model. We therefore indicated by the “question marks” in Fig. 1 that these two assumptions may require further consideration.

### Computational modeling and parameter derivation

Mathematical compartment modeling was performed to represent each compartment in the model using concepts such as mass balance and clearance (used in every module); transit-chain model (used to model the gradient of Ch down the ROS in module 1); Michaelis-Menten kinetics (used to model LDLR-mediated uptake of LDL in the RPE in module 2 and ABCA1-mediated export of Ch in macrophages in module 3); and the theory for transport of particles through cylindrical pores, the Stokes-Einstein relation, and permeability (used to model the permeation of ApoA-I-Ch particles from the BrM to the CC in module 3). Systems of ODEs were solved using MATLAB’s ODE15s solver to arrive at both time-dependent (e.g., in module 1’s turnover process) and steady-state solutions (e.g., modeling the steady-state Ch-content of discs in each transit-chain compartment in module 1, modeling LDLR-mediated influx of LDL into the RPE in module 2, and modeling the steady-state growth of drusen over decades under various model parameters in module 3).

Rather than generating an overall system behavior of the model, our objective in the present work was to analyze each module separately and compare the steady-state flux rates (defined above for each module) to each other. With the exception of the linkage between module 1 (Ch turnover rate) and module 3 (drusen growth rate), the predicted fluxes in each module do not explicitly interact with each other in the current RCD model. Other than drusen growth and regression, no other time-dependent behavior was modeled.

ImageJ software was used to quantify the relative ratio of apical-to-basal ABCA1 protein expression imaged in RPE cells (45). Parameter values and their uncertainties were estimated from experimental data and/or theoretical considerations as detailed in the supplemental material.

## RESULTS

Here we present the Ch flux rates corresponding to the turnover and transport processes represented in the three modules of the RCD model. Details on the derivation of these values are provided in the supplemental material. **Tables 1–3** summarize the flux rates and other key parameters (with their uncertainties) for each of the three modules and indicate the relevant sections of the supplemental material where derivations and references can be found.

**TABLE 1.** Flux rates and other key parameters estimated for module 1 of the RCD model

Flux Rate or Parameter	Definition	Value (Uncertainty %)*^a^*	Unit	Relevant Supplemental Material with Derivation and References
$K_{\mathrm{in}}^{\mathrm{Ch}}$	Steady-state Ch turnover rate (expressed as a Ch flux) corresponds to influx rate of Ch from CC to retina + Ch synthesis rate and is also equal to rate of ROS phagocytosis + Ch uptake by RPE	0.97 (49%) (*f_recycling_* = 1)	pg/mm^2^/min	S1
		5.84 (49%) (*f_recycling_* = 0)		
(See footnote*^b^*)				
$K_{\mathrm{in}}^{\mathrm{discs}}$	Rate of new disc formation entering ROS	85 (6%)	discs/day	S1
*k_t_*	First-order rate constant of disc transfer within ROS transit chain	0.85 (6%)	day^−1^	S1
*k_out_*	First-order rate constant of Ch transferred out of transit compartments 2–10.	0.19 (34%)	day^−1^	S1
*k*_2_	First-order rate constant of Ch recycling from recyclable pool to RIS	Not estimated	day^−1^	S1
*k*_1_	First-order rate constant of Ch uptake from recyclable pool to RPE	Not estimated	day^−1^	S1
*f_recycling_*	Fractional recycling of Ch, equal to *k*_2_/(*k*_1_ + *k*_2_)	Ranges from 0 to 1	Dimensionless	S1

a Derivation of uncertainty estimates is given in the relevant supplemental material.

b These values are rounded to ∼1 and ∼6 in the text.

**TABLE 2.** Flux rates and other key parameters estimated for module 2 of the RCD model

Flux Rate or Parameter	Definition	Value (Uncertainty %)*^a^*	Unit	Relevant Supplemental Material with Derivation and References
$P_{\mathrm{in}}^{\mathrm{LDL}}$	Effective permeability coefficient for the transcytosis of LDL particles from the lumen to the outside of the choroid capillaries.	1.2 × 10^−7^ (7%)	cm/s	S2
$P_{\mathrm{out}}^{\mathrm{LDL}}$	Effective permeability coefficient for the transcytosis of LDL particles from outside the choroid capillaries to inside the lumen.	Assumed to range from 0.12 to 1.2 × 10^−7^	cm/s	S2
$V_{\max}^{\mathrm{LDLR}}/A_{\mathrm{RPE}}$	Maximum LDL uptake rate (expressed as a Ch flux) for an RPE cell that has been downregulated by 90% after a 24 h incubation with LDL protein concentrations (ApoB) exceeding 20 μg/ml	13.1 (50%)	pg/mm^2^/min	S3
$K_{m}^{\mathrm{LDLR}}$	Michaelis parameter for LDLR-mediated uptake (expressed as an LDL-Ch concentration)	5.4 (9%)	mg/dl	S3
$K_{\mathrm{in}}^{\mathrm{ABCA}1}$	ABCA1-mediated transport of Ch from the apical surface of RPE cells to the outer retina (expressed as a Ch flux rate)	6.73 (50%)	pg/mm^2^/min	S4

a Derivation of uncertainty estimates is given in the relevant supplemental material.

**TABLE 3.** Flux rates and other key parameters estimated for module 3 of the RCD model

Flux Rate or Parameter	Definition	Value (Uncertainty %)*^a^*	Unit	Relevant Supplemental Material with Derivation and References
$K_{\mathrm{out}}^{\mathrm{ABCA}1}$	ABCA1-mediated Ch efflux from the basal surface of the RPE to the BrM (expressed as a Ch flux rate)	1.06 (50%)	pg/mm^2^/min	S5
$P_{\mathrm{ApoA}1-\mathrm{Ch}}^{\mathrm{CC}}$	Permeability coefficient of ApoA-I-Ch particles across the CC	1.2 × 10^−4^ (41%)	cm/s	S6
$K_{\mathrm{out}}^{\mathrm{ApoB}-\mathrm{Ch}}$	Ch efflux rate in ApoB containing particles across basal membrane of RPE cells (expressed as a Ch flux rate)	3.0 (50%)	pg/mm^2^/min	S7
$dh_{\mathrm{drusen}}^{\mathrm{growth}}/\mathrm{dt}$	Drusen growth rate in BrM corresponding to Ch turnover rates of 1–6 pg/mm^2^/min (expressed as rate of change of drusen thickness)	0.7 to 4.2 (49%)	μm/year	S8
$K_{m}^{\mathrm{Mac}}$	Michaelis parameter for ABCA1-mediated Ch efflux rate per macrophage (expressed in ApoA-I concentration)	5 (35%)	μg/ml	S9
$v_{\max}^{\mathrm{Mac}}$	Maximum rate of ABCA1-mediated Ch efflux rate per macrophage corresponding to a saturating ApoA-I concentration (expressed per cell)	5.79 × 10^−2^ (27%)	pg/cell/min	S9

a Derivation of uncertainty estimates is given in the relevant supplemental material.

### Module 1: Ch turnover in the ROS

**Fig. 5.**
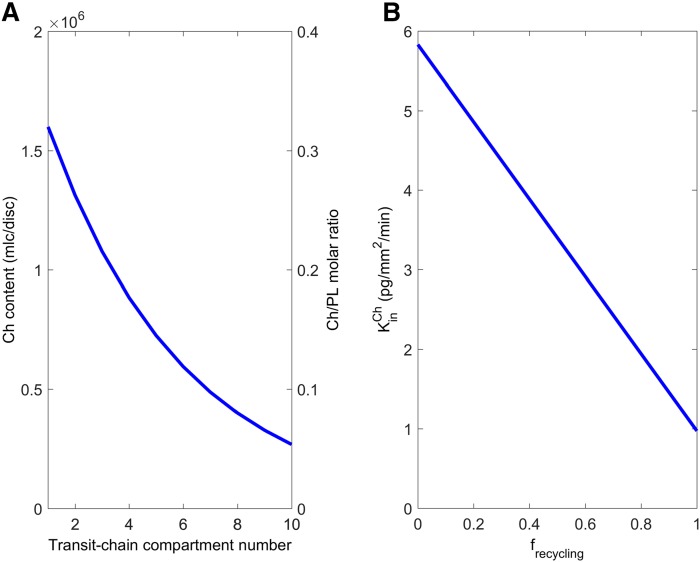
Simulated steady-state behavior of module 1 of the RCD model. A: Ch content of the ROS discs (and their Ch/PL molar ratio) decreases as they transit through the ROS (compartment number 1 corresponds to newly formed discs entering from the RIS and compartment 10 represents the oldest discs that are phagocytosed by the RPE). B: Ch turnover rate, equivalent to the Ch input rate into the RIS $\left(K_{in}^{Ch}\right)$, is predicted to decrease linearly with the fractional recycling of Ch (*f_recycling_*) from the recyclable pool back into the RIS. See supplemental material S1 for further details.

Based on the transit chain model of the ROS discs depicted in Fig. 2, **Fig. 5A** shows the 6-fold decrease in the Ch content per disc along the transit chain, where compartment 1 contains the newly formed discs and compartment 10 contains the oldest discs being phagocytosed by the RPE. The right-hand axis of Fig. 5A displays the corresponding variation in the Ch-to-PL ratio in the discs, which decreases from ∼0.3 to ∼0.05, consistent with the data of Boesze-Battaglia and colleagues (22, 23). Figure 5B shows the steady-state Ch turnover rate derived from the model $\left(K_{in}^{Ch}\right)$, which decreases from ∼6 to ∼1 pg/mm^2^/min as the fractional recycling of Ch from the recyclable pool to the RIS (*f_recycling_*) increases from 0 (no recycling) to 1 (complete recycling) (Table 1).

### Module 2: delivery of Ch from CC to RPE to the outer retina

#### Step 1: transcytosis from CC to BrM.

As depicted in Fig. 3, the first step in delivering systemic LDL-Ch to the outer retina occurs via transcytotic vesicular transport across the CC endothelium into the BrM. In supplemental material S2, the effective transcytotic permeability of LDL across the capillary endothelium from inside the lumen to outside $\left(P_{in}^{LDL}\right)$ is estimated to be ∼1.2 × ∼1.2 × 10^−7^ cm/s (Table 2). We assume the effective permeability of LDL from outside the lumen to inside $\left(P_{out}^{LDL}\right)$ is between 0.1 and 1 times $P_{in}^{LDL}$.

#### Step 2: LDLR-mediated endocytosis by the RPE.

**Fig. 6.**
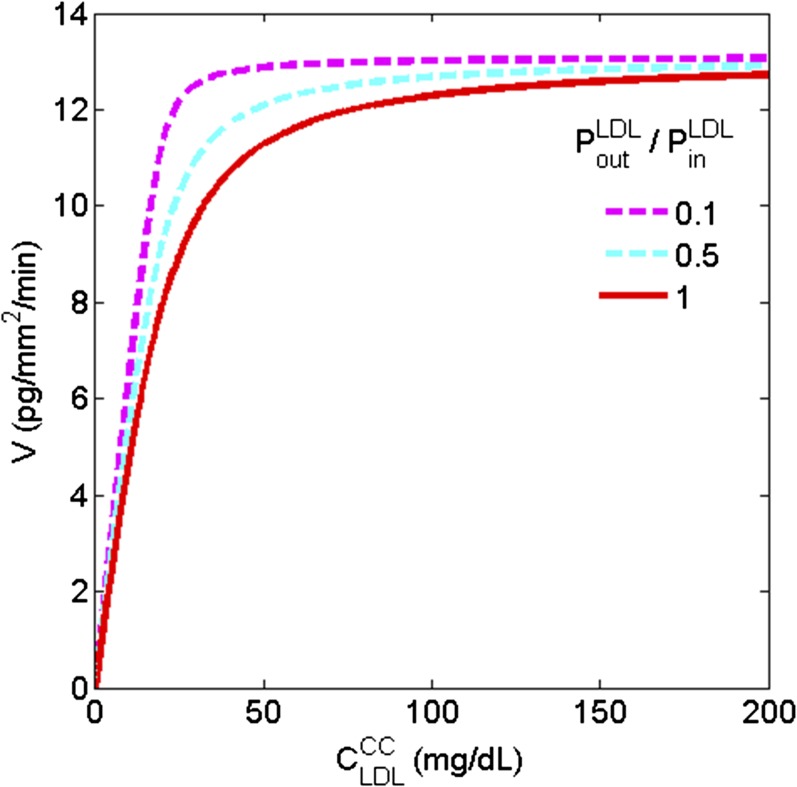
Dependence of the LDLR-mediated Ch uptake rate by the RPE, expressed as a Ch flux (V), as a function of the LDL-Ch concentration in the choroidal capillary bed $\left(C_{LDL}^{CC}\right)$ for values of the transcytosis permeability ratio $\;P_{out}^{LDL}/P_{in}^{LDL}$ equal to 0.1, 0.5 and 1. See supplemental material S3 for further details.

Combining the transcytotic pathway with the LDLR-mediated endocytosis pathway by the RPE (using the differential equation given in supplemental equation S3.2 of supplemental material S3), we show in **Fig. 6** the relationship between the steady-state Ch uptake by LDLR and the LDL-Ch concentration in the CC for $P_{out}^{LDL}/P_{in}^{LDL}$ equal to 0.1, 0.5, and 1. In all three cases the maximal flux rate approaches 13.1 pg Ch/mm^2^/min, which is comparable to the previously estimated Ch turnover rates in the ROS (Table 2). Moreover, the LDL-Ch levels at which 90% of the maximum flux is achieved are approximately 24, 43, and 67 mg/dl, respectively; in all cases lower than normal physiological serum levels (∼100 mg/dl LDL-Ch).

#### Step 3: ABCA1-mediated transport from RPE to outer retina.

As detailed in supplemental material S4, the rate of ABCA1-mediated transport of Ch from RPE cells to the outer retina $\left(K_{in}^{ABCA1}\right)$ was estimated to be 6.73 pg/mm^2^/min, also comparable in magnitude to the previously estimated Ch turnover rates in the ROS (Table 2).

### Module 3: efflux of Ch from RPE to BrM to CC (including rates of drusen growth and clearance)

#### ABCA1-mediated efflux out of the RPE.

From an analysis of the polarized ABCA1 expression reported on mouse RPE cells (45), we found the apical side of the RPE to have 6.37 times higher ABCA1 expression than the basal side (supplemental material S5). Using this factor to scale the prior estimate of $K_{in}^{ABCA1}$, we estimate $K_{out}^{ABCA1}$ to be ∼1.1 pg/mm^2^/min, which is comparable to the lower limit of Ch turnover rate in the ROS (Table 3).

The ApoA-I-Ch particles that result from ABCA1-mediated Ch efflux from RPE cells or from macrophages in the BrM are small enough to pass through the diaphragmed fenestra of the CC endothelium and be cleared into the systemic circulation. As described in supplemental material S6, we have estimated the permeability coefficient of ApoA-I-Ch across the CC endothelium to be 1.2 × 10^−4^ cm/s (Table 3). Assuming the flux rate of ApoA-I-Ch (expressed in Ch mass) ranges from the previous estimate of ∼1 pg/mm^2^/min to the 6-fold higher limit for $K_{in}^{Ch}$, the corresponding concentrations of ApoA-I-Ch in the BrM will range from 0.0014 to 0.0084 mg Ch/dl or from ∼0.01 to ∼0.06 mg ApoA-I/dl.

#### ApoB-Ch particle secretion out of the RPE.

As detailed in supplemental material S7, if ApoB secretion in the RPE was identical to the hepatocyte, the Ch efflux rate of 70 nm ApoB-Ch particles from RPE to the BrM, $K_{out}^{ApoB\text{-}Ch}$, would be 967 pg/mm^2^/min, two orders of magnitude larger than the maximal Ch turnover rate in the ROS. Because the RPE expresses much lower mRNA levels of ApoB (7.5%) and MTP-A (4%) compared with liver (54), we have scaled this value by 0.003 (= 0.075 × 0.04) to obtain a more realistic estimate of $K_{out}^{ApoB\text{-}Ch}$ of ∼3 pg/mm^2^/min (Table 3), which is also comparable to the range of estimated Ch turnover rates $K_{in}^{Ch}$.

#### Ch deposition in BrM (rate of drusen growth).

**Fig. 7.**
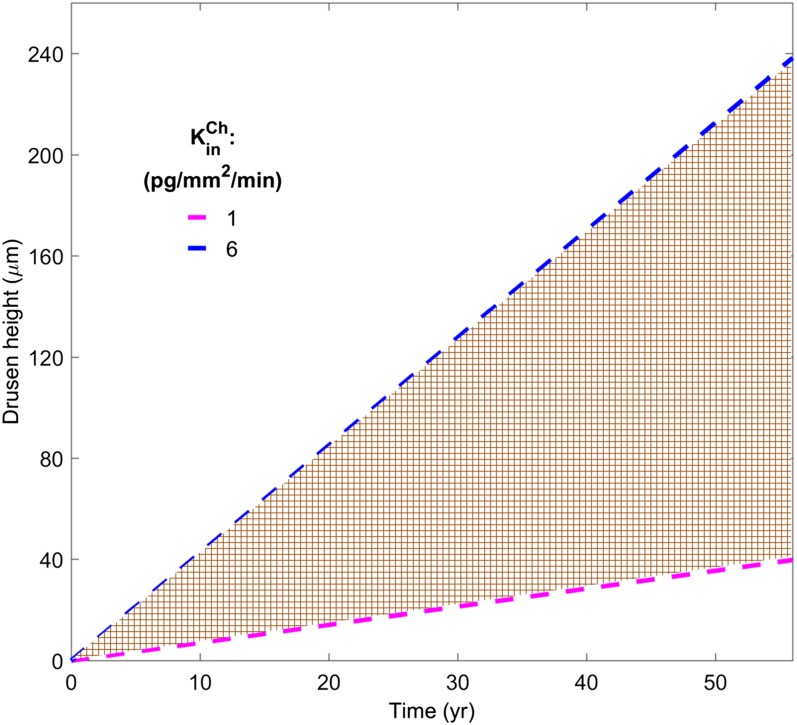
The linear rate of increase of drusen height (including basal linear deposits) over 50 years assuming that all of the Ch efflux from the RPE occurs via large ApoB-containing particles that are deposited into the BrM. The blue and purple dashed lines correspond to the maximum and minimum efflux rates, i.e., the Ch turnover rates, $K_{in}^{Ch}$ = 6 and 1 pg/mm^2^/min, respectively. The brown hatched region corresponds to intermediate Ch turnover rates, which depend on the recycling fraction in the ROS. See supplemental material S8 for further details.

Based on a number of assumptions, the amount of Ch deposited in the BrM (corresponding to the drusen height) will increase linearly with time, with a slope proportional to $K_{out}^{ApoB\text{-}Ch}$ (see supplemental material S8). For values of $K_{out}^{ApoB\text{-}Ch}$ associated with the previously estimated range of $K_{in}^{Ch}$ (∼1–6 pg/mm^2^/min), the corresponding drusen growth rate, $dh_{drusen}^{growth}/dt$, is estimated to be ∼0.7–4.2 μm/year (Table 3). As illustrated in **Fig. 7**, if such growth rates persist for five decades, the resulting thickness of Ch deposited in the BrM, i.e., drusen height would range from 35 to 210 μm.

#### Macrophage-mediated clearance of drusen.

As described in supplemental material S9, the three main determinants of the macrophage-mediated drusen clearance rate $\left(dh_{Drusen}^{Mac}/dt\right)$ are: macrophage density $\left(C_{retina}^{Mac}\right)$, macrophage ABCA1 activity $\;\left(v_{max}^{Mac}\right)$, and the lipid-free ApoA-I concentration in the BrM proximal to the macrophage(s) $\left(C_{BrM}^{ApoA\text{-}I}\right)$.

Based on Penfold’s histological data in patients with varying stages of progressive AMD and other theoretical considerations (55), we estimate that the macrophage density in a 100 μm-thick region of BrM containing a large drusen could conceivably range from 0 to 5,000 cells/mm^2^ (see supplemental material S9 for data and calculations).

**Fig. 8.**
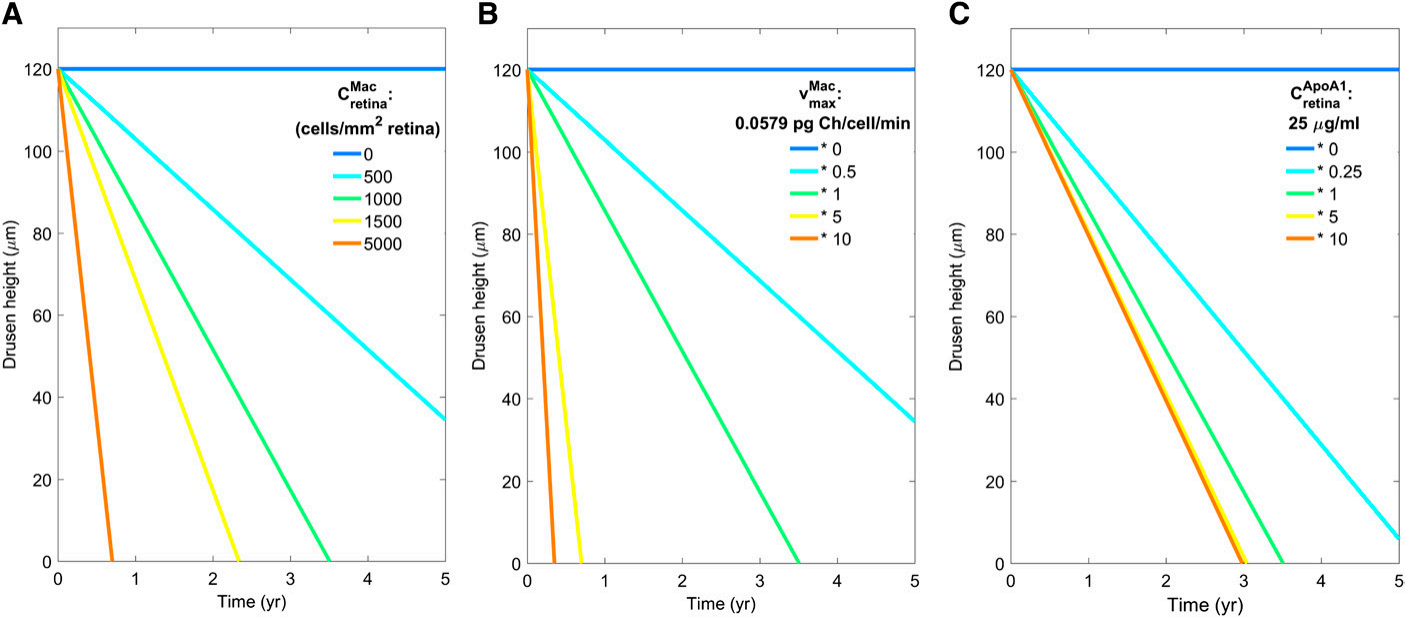
Dependence of drusen clearance rate (rate of decrease in drusen thickness) on macrophage density in the BrM from 0 to 5,000 cells/mm^2^ (A); maximum capacity of ABCA1-mediated efflux per macrophage $\left(v_{max}^{Mac}\right)$ with fold-changes varying from 0 to 10 (B); lipid-poor ApoA-I concentration in vicinity of drusen $\left(C_{BrM}^{ApoA\text{-}I}\right)$ with fold changes varying from 0 to 10 (C). The baseline values in the simulations (corresponding to green lines) are: $C_{retina}^{Mac}$ = 1,000 cells/mm^2^; $v_{max}^{Mac}$ = 0.0579 pg/cell/min; and $C_{BrM}^{ApoA-I}$ = 25 μg/ml. See supplemental material S9 for further details.

**Figure 8A** shows a simulation of the decrease in drusen height for macrophage densities ranging from 0 to 5,000 cells/mm^2^. In order to compare the simulations with serial optical coherence tomography data reported by Ouyang et al. (9) the initial drusen thickness was taken to be 120 μm.The time to fully clear the 120 μm drusen is seen to be more than 5 years with a density of 500 cells/mm^2^ and decreases to less than 1 year with a density of 5,000 cells/mm^2^. In the patient of Ouyang et al. (9) with extremely rapid clearance, the time for disappearance of the drusen was 10 months and would correspond to an estimated macrophage density of ∼3,500 cells/mm^2^.

Figure 8B shows a simulation of the theoretical effect of varying $v_{max}^{Mac}$ on the drusen clearance rate. With increasing ABCA1 expression, the time to clear a large drusen decreases markedly. In contrast, increasing $C_{BrM}^{ApoA\text{-}I}$ is shown in Fig. 8C to reach a limiting value of about 3 years due to the saturation of the ABCA1-transport mechanism, i.e., at levels that exceed the Michaelis parameter for ABCA1-mediated Ch efflux $K_{m}^{Mac}$ (Table 3). Conversely, if the level of $C_{BrM}^{ApoA\text{-}I}$ is much lower than the assumed baseline level (fold-change of 0.25), the rate of macrophage-mediated drusen clearance will be much slower.

## DISCUSSION

### Key findings of the RCD model

As summarized in Tables 1–3, we have found that the estimated rates of Ch turnover in the ROS $\left(K_{in}^{Ch}\right)$, LDLR-mediated endocytosis by the RPE $\left(V_{max}^{LDLR}\right)$, ABCA1-mediated transport from the RPE to the outer retina $\left(K_{in}^{ABCA1}\right)$, ABCA1-mediated efflux out of the RPE $\left(K_{out}^{ABCA1}\right)$, and ApoB-Ch particle secretion out of the RPE $\left(K_{out}^{ApoB\text{-}Ch}\right)$, when expressed as Ch fluxes, are all of the same order of magnitude, ranging from 1 to 13 pg/mm^2^/min. The consistency of these fluxes suggests that the processes of Ch delivery from the CC to the outer retina and the reverse processes of Ch efflux from the RPE to the BrM are well designed and/or regulated to match the rate at which Ch is needed in the formation of Ch-rich ROS discs. Furthermore our estimate of the range of drusen growth rates, 0.7–4.2 μm/year, is consistent with clinical observations on the increasing prevalence of large (100 μm) drusen with increasing age (3). Lastly, our estimate of the drusen clearance rate (Fig. 8), while based on limited data on macrophage density in the BrM, nevertheless shows that the rapid clearance of drusen seen in some patients (9) may be explained by a mechanism of ABCA1-mediated macrophage efflux.

### Questions that motivated the development of the RCD model

#### What is the physiological rate of Ch turnover in the outer retina, and what mechanisms govern it?

The RCD model estimates the Ch turnover rate in the human retina to range from ∼1 to ∼6 pg/mm^2^/min. The 6-fold variation reflects the extent to which Ch that is effluxed from the ROS discs during their 11 day transit through the ROS recycles back to the RIS where new discs are formed. This process is quantified in the RCD model by the recycling fraction, which varies from 0 (no-recycling) to 1 (complete recycling). As the recycling fraction increases, the Ch turnover rate decreases. Although several sources have discussed the potential role of Ch recycling in the retina (13, 57), the transport mechanisms responsible for Ch removal and recycling are not known. Fliesler and Bretillion (13) have suggested that ApoB, ApoA-I, and CETP may be involved.

#### How is Ch provided to the outer retina (via delivery by lipoproteins versus local synthesis)?

The RCD model suggests that Ch is delivered from the CC to the RPE cells by LDLR-mediated uptake in the basal membrane and could be transported to the RIS by an ABCA1-mediated process in the apical membrane at a sufficient rate to satisfy the Ch turnover requirements in the ROS. Thus, Ch synthesis is not required. Our calculations (Fig. 6) suggest that LDLR-mediated uptake by RPE cells is saturated at very low LDL-Ch levels in the CC. This may explain why elevated plasma LDL-Ch levels have not been consistently found to increase the risk for AMD (58–60) and why treatment of dry AMD patients with statins appears to have relatively little effect on the disease (61, 62). The recent study on high-dose statins by Vavvas et al. (63), while showing improvement in some clinical features of AMD, suggests that the mechanism is not due solely to lowering of LDL-Ch levels. Conversely the role of serum LDL-Ch as the main source of Ch in the outer retina may explain the pathogenesis of certain forms of retinitis pigmentosa that occur in severe LDL-deficiency states (64–67).

In contrast to our interpretation, we note that Lin et al. (68) have recently reported that local Ch synthesis in the mouse retina contributes about 70% of the retinal Ch turnover rate. This finding could be specific to the mouse and other species that lack CETP, whose role in retinal Ch metabolism has been suggested by Tserentsoodol et al. (14) and Fliesler and Bretillon (13). As shown in supplemental material S10, the Ch turnover rate in the mouse reported by Lin et al. (68) (21 μg/g wet tissue/day) corresponds to a value of 2.9 pg/mm^2^/min and the total Ch content of the retina (1.13 mg/g wet tissue) corresponds to a level of 226 ng/mm^2^. Taking account of the different dimensions and density of the ROS in mice compared with humans and primates, we estimate the Ch turnover in the outer retina of the mouse to range from 3.7 to 22 pg/mm^2^/min depending on the recyclable fraction and the Ch content to be 126 ng/mm^2^ (see supplemental material S10). Thus, our model-based estimates of Ch turnover and Ch content in the mouse are in reasonable agreement with Lin’s experimental findings, despite the fact that, in the mouse, Ch synthesis appears to be the main source of retinal Ch.

#### How does Ch leave the outer retina?

The RCD model quantifies two pathways for Ch efflux from the RPE. The first pathway, which is presumably the normal physiological mechanism, is ABCA1-mediated Ch efflux from the basal membrane to small lipid-poor ApoA-I in the BrM. The second pathway, which Curcio and colleagues suggest as a pathological mechanism (3, 12, 15–17), involves the secretion of large (∼70 nm) ApoB-containing particles loaded with cholesterol ester, which are presumed to be entrapped in the BrM first as basal linear deposits and then as drusen. Our flux calculations suggest that the first pathway may have limited capacity to efflux all of the Ch entering the RPE from the ROS and outer retina. Therefore for individuals with a high Ch turnover rate (low recycling fraction) or a low expression of ABCA1 on the basal membrane, the capacity of the first pathway will be exceeded and the second pathway involving large ApoB-Ch particles will be needed to efflux Ch. The mechanisms that control the interplay of these two pathways have not been elucidated in the RPE. A role for oxysterols has been recently proposed in retinal Ch metabolism (69) and could conceivably be involved in modulating these pathways.

#### What determines the slow rate of drusen growth in dry AMD?.

The RCD model predicts that the drusen growth rate will vary from 0.7 to 4.2 μm/year, depending on the flux rate of large ApoB-Ch particles (∼1–6 pg/mm^2^/min) which, as noted above, will depend on the recycling fraction. In subjects with an ApoB-Ch particle flux of ∼6 pg/mm^2^/min, a large (100 μm thick) drusen may appear in 25 years. Individuals with a lower ApoB-Ch particle flux, e.g., ∼3 pg/mm^2^/min, will require 50 years or more.

#### What processes are responsible for the rapid rates of drusen regression observed in some patients?.

The RCD model predicts that the rate of macrophage-mediated drusen clearance will depend on the macrophage density in the BrM, the macrophage ABCA1 activity, and the lipid-poor ApoA-I concentration in the vicinity of the macrophages. Our calculations suggest that this mechanism can explain the very rapid clearance of a large drusen in the patient studied by Ouyang et al. (9), if the macrophage density is about 3,500 cells/mm^2^. This value exceeds the maximum mean leukocyte density reported by Penfold (55), but is well below theoretical limit of 15,000 cells/mm^2^ based on the packing of spheres. More longitudinal data on drusen clearance rates and macrophage densities are needed to clarify this issue.

### Potential limitations of the model

Many of the RCD model calculations were derived indirectly from data on hepatic lipoprotein metabolism and scaled by geometric considerations or with limited in vitro data on RPE cells. In the case of the Ch turnover rate, the calculations were based more directly on ROS turnover data from primates and extensive biochemical analysis of ROS disc composition in other species (see supplemental material S1 for references). Direct measurements of Ch turnover and flux rates corresponding to the mechanisms in our model would be valuable in confirming some of the key assumptions and results. This is particularly true for the hypothesized roles of ABCA1-mediated Ch efflux from the apical and basal membranes of the RPE. While ABCA1 expression has been demonstrated in the macular region of the RPE in the monkey (19) and appears to be greater on the apical than basal membrane in the mouse (45), experimental measurements of ABCA1-mediated Ch efflux from the apical surface to the outer retina have not yet been reported (70).

A further limitation of the current model is the lack of knowledge about the mechanisms involved in the ROS recycling compartment. While ApoB, ApoA-I, and CETP have been located to the ROS layer (13), their functions and potential roles with respect to Ch recycling remain unclear. Furthermore, the size of the recycling compartment (which cannot be estimated from the presently available data) and its location in the outer retina have not been determined. The interplay between the two pathways of Ch efflux from the RPE to the BrM (via small ApoA-I-Ch particles and large ApoB-Ch particles) is also missing from the current model and should be further developed along the lines of models of Ch metabolism in the liver (71, 72).

Finally, the RCD model focuses on the outer retina and does not consider the interchange between the outer and inner layers, nor does it consider the role of cone cells in the Ch dynamics of the foveal region of the macula. Future development of the RCD model may incorporate these components and address the inter-subject variation in parameters and the effects of aging as additional experimental data become available.

### Human genetic findings in relation to the RCD model

In the largest AMD genome-wide association study to date (73), associations have been reported for LIPC, CETP, ABCA1, and APOE, thereby providing evidence that lipid genes are important in retinal function and that germline mutations in lipid loci affect the development of AMD later in life. Another recent study (74) found that the presence of a rare CETP missense mutation (rs2303790), almost exclusively present in East Asians, increased the risk of AMD by 70%, suggesting a critical role of CETP in retinal lipid metabolism. While the RCD model does not specify the function of CETP in the retina, immunohistochemistry studies from the monkey retina (14) have shown the clear presence of CETP protein in the photoreceptor layer. Based on this finding and the role of CETP in transferring cholesterol esters between lipoprotein particles (in the circulation), it is conceivable that CETP plays a necessary role in Ch recycling in the outer retina. Thus, impaired CETP function in the context of the RCD model would reduce Ch recycling and increase Ch turnover and Ch efflux via the ApoB pathway, thereby increasing the risk for developing AMD.

Clinical case reports also highlight the ophthalmic effects of systemic Ch disorders and may be considered in relation to the RCD model. Cases of ApoB deficiency in hypobetalipoproteinemia and abetalipoproteinemia (64–67) result in retinitis pigmentosa. In the context of module 2 of the RCD model, ApoB deficiency would markedly reduce Ch uptake by the RPE, resulting in impaired ROS disc formation and function. Defective ABCA1-mediated transport in Tangier’s disease (75, 76) and ApoA-I deficiency (77) are likewise associated with retinal pathologies that could result from impaired Ch transport within or out of the outer retina as represented in modules 2 and 3 of the RCD model.

### Insights into the pathophysiology of dry AMD

The key pathophysiological insights that we derive from the RCD model concern the linkages between the Ch turnover rate, the recycling fraction, the extent of Ch efflux via the ApoB-Ch particle pathway and the dynamics of drusen growth. Our model suggests that retinal Ch turnover and recycling in the ROS are central to understanding the normal physiology of RCD and the slow rate of Ch deposition in dry AMD. This analysis provides quantitative support for the earlier conjecture of Young (11) that drusen are derived from RPE elimination products resulting from the phagocytosis of ROS discs and provides strong support for Curcio’s (3, 12, 15–17) identification of the role of large ApoB-Ch particles in this process.

We further note that our analysis of drusen clearance suggests that very high densities of macrophages are needed to account for the rapid clearance of drusen seen in some patients. One could speculate that the secretion of various cytokines by macrophages, including VEGF, complement factors, and other inflammatory mediators, could produce damage to the overlying RPE structure leading to geographic atrophy and/or play a role in the neovascularization process. Efforts to quantify the macrophage density and characterize the macrophage types in human drusen would help us link the RCD model to a comprehensive mechanistic description of wet AMD and geographic atrophy.

In conclusion, we have developed a quantitative model of retinal Ch dynamics based on a variety of experimental data and theoretical concepts in the literature. We have used this model to generate new hypotheses about the mechanisms of Ch turnover in the outer retina, the slow rate of drusen formation, and the role of macrophages in drusen clearance. Further experimental studies are needed to test these hypotheses and enable refinement of the RCD model.

## Supplementary Material

Supplemental Data

## References

[1] Albert A. D., and Boesze-Battaglia K. 2005 The role of cholesterol in rod outer segment membranes. Prog. Lipid Res. 44: 99–124. 15924998 10.1016/j.plipres.2005.02.001 PMC4732711

[2] Young R. W. 1971 The renewal of rod and cone outer segments in the rhesus monkey. J. Cell Biol. 49: 303–318. 19866760 10.1083/jcb.49.2.303 PMC2108322

[3] Curcio C. A., Millican C. L., Bailey T., and Kruth H. S. 2001 Accumulation of cholesterol with age in human Bruch’s membrane. Invest. Ophthalmol. Vis. Sci. 42: 265–274. 11133878

[4] Haimovici R., Gantz D. L., Rumelt S., Freddo T. F., and Small D. M. 2001 The lipid composition of drusen, Bruch’s membrane, and sclera by hot stage polarizing light microscopy. Invest. Ophthalmol. Vis. Sci. 42: 1592–1599. 11381066

[5] Wang L., Clark M. E., Crossman D. K., Kojima K., Messinger J. D., Mobley J. A., and Curcio C. A. 2010 Abundant lipid and protein components of drusen. PLoS One. 5: e10329. 20428236 10.1371/journal.pone.0010329 PMC2859054

[6] Holz F. G., Sheraidah G., Pauleikhoff D., and Bird A. C. 1994 Analysis of lipid deposits extracted from human macular and peripheral Bruch’s membrane. Arch. Ophthalmol. 112: 402–406. 8129668 10.1001/archopht.1994.01090150132035

[7] Yehoshua Z., Wang F., Rosenfeld P. J., Penha F. M., Feuer W. J., and Gregori G. 2011 Natural history of drusen morphology in age-related macular degeneration using spectral domain optical coherence tomography. Ophthalmology. 118: 2434–2441. 21724264 10.1016/j.ophtha.2011.05.008 PMC3189426

[8] van de Ven J. P., Boon C. J., Smailhodzic D., Lechanteur Y. T., den Hollander A. I., Hoyng C. B., and Theelen T. 2012 Short-term changes of Basal laminar drusen on spectral-domain optical coherence tomography. Am. J. Ophthalmol. 154: 560–567. 22626619 10.1016/j.ajo.2012.03.012

[9] Ouyang Y., Heussen F. M., Hariri A., Keane P. A., and Sadda S. R. 2013 Optical coherence tomography-based observation of the natural history of drusenoid lesion in eyes with dry age-related macular degeneration. Ophthalmology. 120: 2656–2665. 23830761 10.1016/j.ophtha.2013.05.029 PMC5340146

[10] Toy B. C., Krishnadev N., Indaram M., Cunningham D., Cukras C. A., Chew E. Y., and Wong W. T. 2013 Drusen regression is associated with local changes in fundus autofluorescence in intermediate age-related macular degeneration. Am. J. Ophthalmol. 156: 532–542.e1. 23830564 10.1016/j.ajo.2013.04.031 PMC3748172

[11] Young R. W. 1987 Pathophysiology of age-related macular degeneration. Surv. Ophthalmol. 31: 291–306. 3299827 10.1016/0039-6257(87)90115-9

[12] Curcio C. A., Messinger J. D., Sloan K. R., McGwin G., Medeiros N. E., and Spaide R. F. 2013 Subretinal drusenoid deposits in non-neovascular age-related macular degeneration: morphology, prevalence, topography, and biogenesis model. Retina. 33: 265–276. 23266879 10.1097/IAE.0b013e31827e25e0 PMC3870202

[13] Fliesler S. J., and Bretillon L. 2010 The ins and outs of cholesterol in the vertebrate retina. J. Lipid Res. 51: 3399–3413. 20861164 10.1194/jlr.R010538 PMC2975712

[14] Tserentsoodol N., Gordiyenko N. V., Pascual I., Lee J. W., Fliesler S. J., and Rodriguez I. R. 2006 Intraretinal lipid transport is dependent on high density lipoprotein-like particles and class B scavenger receptors. Mol. Vis. 12: 1319–1333. 17110915

[15] Curcio C. A., Presley J. B., Malek G., Medeiros N. E., Avery D. V., and Kruth H. S. 2005 Esterified and unesterified cholesterol in drusen and basal deposits of eyes with age-related maculopathy. Exp. Eye Res. 81: 731–741. 16005869 10.1016/j.exer.2005.04.012

[16] Curcio C. A., Johnson M., Huang J-D., and Rudolf M. 2009 Aging, age-related macular degeneration, and the response-to-retention of apolipoprotein B-containing lipoproteins. Prog. Retin. Eye Res. 28: 393–422. 19698799 10.1016/j.preteyeres.2009.08.001 PMC4319375

[17] Curcio C. A., Johnson M., Huang J-D., and Rudolf M. 2010 Apolipoprotein B-containing lipoproteins in retinal aging and age-related macular degeneration. J. Lipid Res. 51: 451–467. 19797256 10.1194/jlr.R002238 PMC2817575

[18] Sene A., Khan A. A., Cox D., Nakamura R. E., Santeford A., Kim B. M., Sidhu R., Onken M. D., Harbour J. W., and Hagbi-Levi S. 2013 Impaired cholesterol efflux in senescent macrophages promotes age-related macular degeneration. Cell Metab. 17: 549–561. 23562078 10.1016/j.cmet.2013.03.009 PMC3640261

[19] Chen J., and Smith L. E. 2013 Altered cholesterol homeostasis in aged macrophages linked to neovascular macular degeneration. Cell Metab. 17: 471–472. 23562072 10.1016/j.cmet.2013.03.010 PMC3669899

[20] Young R. W., and Bok D. 1969 Participation of the retinal pigment epithelium in the rod outer segment renewal process. J. Cell Biol. 42: 392–403. 5792328 10.1083/jcb.42.2.392 PMC2107669

[21] Young R. W. 1967 The renewal of photoreceptor cell outer segments. J. Cell Biol. 33: 61–72. 6033942 10.1083/jcb.33.1.61 PMC2107286

[22] Boesze-Battaglia K., Hennessey T., and Albert A. D. 1989 Cholesterol heterogeneity in bovine rod outer segment disk membranes. J. Biol. Chem. 264: 8151–8155. 2722776 PMC4720381

[23] Boesze-Battaglia K., Fliesler S. J., and Albert A. D. 1990 Relationship of cholesterol content to spatial distribution and age of disc membranes in retinal rod outer segments. J. Biol. Chem. 265: 18867–18870. 2229047 PMC4471995

[24] Corless J. M. 2012 Cone outer segments: a biophysical model of membrane dynamics, shape retention, and lamella formation. Biophys. J. 102: 2697–2705. 22735519 10.1016/j.bpj.2012.04.052 PMC3379012

[25] Shirinifard A., Glazier J. A., Swat M., Gens J. S., Family F., Jiang Y., and Grossniklaus H. E. 2012 Adhesion failures determine the pattern of choroidal neovascularization in the eye: a computer simulation study. PLOS Comput. Biol. 8: e1002440. 22570603 10.1371/journal.pcbi.1002440 PMC3342931

[26] Family F., Mazzitello K., Arizmendi C., and Grossniklaus H. 2010 Statistical physics of age related macular degeneration. Phys. Procedia. 4: 21–33.

[27] Savic R. M., Jonker D. M., Kerbusch T., and Karlsson M. O. 2007 Implementation of a transit compartment model for describing drug absorption in pharmacokinetic studies. J. Pharmacokinet. Pharmacodyn. 34: 711–726. 17653836 10.1007/s10928-007-9066-0

[28] Tserentsoodol N., Sztein J., Campos M., Gordiyenko N. V., Fariss R. N., Lee J. W., Fliesler S. J., and Rodriguez I. R. 2006 Uptake of cholesterol by the retina occurs primarily via a low density lipoprotein receptor-mediated process. Mol. Vis. 12: 1306–1318. 17110914

[29] Hayes K. C., Lindsey S., Stephan Z., and Brecker D. 1989 Retinal pigment epithelium possesses both LDL and scavenger receptor activity. Invest. Ophthalmol. Vis. Sci. 30: 225–232. 2536645

[30] Bazan N. G., Gordon W. C., and de Turco E. B. R. 1992 Docosahexaenoic acid uptake and metabolism in photoreceptors: retinal conservation by an efficient retinal pigment epithelial cell-mediated recycling process. *In* Bazan N. G., Murphy M. G., and Toffano G., editors. Springer, New York 295–306. 10.1007/978-1-4615-3426-6_26 1386177

[31] Wang N., and Anderson R. E. 1993 Transport of 22: 6n-3 in the plasma and uptake into retinal pigment epithelium and retina. Exp. Eye Res. 57: 225–233. 8405189 10.1006/exer.1993.1118

[32] Elner V. M. 2002 Retinal pigment epithelial acid lipase activity and lipoprotein receptors: effects of dietary omega-3 fatty acids. Trans. Am. Ophthalmol. Soc. 100: 301–338. 12545699 PMC1358968

[33] Miller J. W., Walsh A. W., Kramer M., Hasan T., Michaud N., Flotte T. J., Haimovici R., and Gragoudas E. S. 1995 Photodynamic therapy of experimental choroidal neovascularization using lipoprotein-delivered benzoporphyrin. Arch. Ophthalmol. 113: 810–818. 7540388 10.1001/archopht.1995.01100060136048

[34] Haimovici R., Kramer M., Miller J. W., Hasan T., Flotte T. J., Schomacker K. T., and Gradoudas E. S. 1997 Localization of lipoprotein-delivered benzoporphyrin derivative in the rabbit eye. Curr. Eye Res. 16: 83–90. 9068937 10.1076/ceyr.16.2.83.5088

[35] Gordiyenko N., Campos M., Lee J. W., Fariss R. N., Sztein J., and Rodriguez I. R. 2004 RPE cells internalize low-density lipoprotein (LDL) and oxidized LDL (oxLDL) in large quantities in vitro and in vivo. Invest. Ophthalmol. Vis. Sci. 45: 2822–2829. 15277509 10.1167/iovs.04-0074

[36] Jeyarajah E. J., Cromwell W. C., and Otvos J. D. 2006 Lipoprotein particle analysis by nuclear magnetic resonance spectroscopy. Clin. Lab. Med. 26: 847–870. 17110242 10.1016/j.cll.2006.07.006

[37] Mora S., Otvos J. D., Rifai N., Rosenson R. S., Buring J. E., and Ridker P. M. 2009 Lipoprotein particle profiles by nuclear magnetic resonance compared with standard lipids and apolipoproteins in predicting incident cardiovascular disease in women. Circulation. 119: 931–939. 19204302 10.1161/CIRCULATIONAHA.108.816181 PMC2663974

[38] Sarin H. 2010 Physiologic upper limits of pore size of different blood capillary types and another perspective on the dual pore theory of microvascular permeability. J. Angiogenes. Res. 2: 14. 20701757 10.1186/2040-2384-2-14 PMC2928191

[39] Brown M. S., and Goldstein J. L. 1979 Receptor-mediated endocytosis: insights from the lipoprotein receptor system. Proc. Natl. Acad. Sci. USA. 76: 3330–3337. 226968 10.1073/pnas.76.7.3330 PMC383819

[40] Frank P. G., Pavlides S., and Lisanti M. P. 2009 Caveolae and transcytosis in endothelial cells: role in atherosclerosis. Cell Tissue Res. 335: 41–47. 18688651 10.1007/s00441-008-0659-8

[41] von Eckardstein A., and Rohrer L. 2009 Transendothelial lipoprotein transport and regulation of endothelial permeability and integrity by lipoproteins. Curr. Opin. Lipidol. 20: 197–205. 19395962 10.1097/MOL.0b013e32832afd63

[42] Dehouck B., Fenart L., Dehouck M-P., Pierce A., Torpier G., and Cecchelli R. 1997 A new function for the LDL receptor: transcytosis of LDL across the blood–brain barrier. J. Cell Biol. 138: 877–889. 9265653 10.1083/jcb.138.4.877 PMC2138047

[43] Brown M. S., and Goldstein J. L. 1975 Regulation of the activity of the low density lipoprotein receptor in human fibroblasts. Cell. 6: 307–316. 212203 10.1016/0092-8674(75)90182-8

[44] Harwood H. J., and Pellarin L. D. 1997 Kinetics of low-density lipoprotein receptor activity in Hep-G2 cells: derivation and validation of a Briggs-Haldane-based kinetic model for evaluating receptor-mediated endocytotic processes in which receptors recycle. Biochem. J. 323: 649–659. 9169597 10.1042/bj3230649 PMC1218367

[45] Ananth S., Gnana-Prakasam J. P., Bhutia Y. D., Veeranan-Karmegam R., Martin P. M., Smith S. B., and Ganapathy V. 2014 Regulation of the cholesterol efflux transporters ABCA1 and ABCG1 in retina in hemochromatosis and by the endogenous siderophore 2,5-dihydroxybenzoic acid. Biochim. Biophys. Acta. 1842: 603–612. 24462739 10.1016/j.bbadis.2014.01.010 PMC4289134

[46] Bungay P. M., and Brenner H. 1973 The motion of a closely fitting sphere in a fluid-filled tube. Int. J. Multiphase Flow. 1: 25–56.

[47] Deen W. M., Bridges C. R., Brenner B. M., and Myers B. D. 1985 Heteroporous model of glomerular size selectivity: application to normal and nephrotic humans. Am. J. Physiol. 249: F374–F389. 4037090 10.1152/ajprenal.1985.249.3.F374

[48] Deen W. M. 1987 Hindered transport of large molecules in liquid-filled pores. AIChE J. 33: 1409–1425.

[49] Stylianopoulos T., Soteriou K., Fukumura D., and Jain R. K. 2013 Cationic nanoparticles have superior transvascular flux into solid tumors: insights from a mathematical model. Ann. Biomed. Eng. 41: 68–77. 22855118 10.1007/s10439-012-0630-4 PMC3886728

[50] Ishibashi R., Sugita A., and Yoshioka H. 1984 Dynamic changes of fenestrations in choriocapillaries. Kurume Med. J. 31: 309–315. 6543894 10.2739/kurumemedj.31.309

[51] Melamed S., Ben-Sira I., and Ben-Shaul Y. 1980 Ultrastructure of fenestrations in endothelial choriocapillaries of the rabbit–a freeze-fracturing study. Br. J. Ophthalmol. 64: 537–543. 7426569 10.1136/bjo.64.7.537 PMC1043755

[52] Johnson M., Dabholkar A., Huang J-D., Presley J. B., Chimento M. F., and Curcio C. A. 2007 Comparison of morphology of human macular and peripheral Bruch’s membrane in older eyes. Curr. Eye Res. 32: 791–799. 17882712 10.1080/02713680701550660 PMC2562033

[53] Wang L., Li C-M., Rudolf M., Belyaeva O. V., Chung B. H., Messinger J. D., Kedishvili N. Y., and Curcio C. A. 2009 Lipoprotein particles of intraocular origin in human Bruch membrane: an unusual lipid profile. Invest. Ophthalmol. Vis. Sci. 50: 870–877. 18806290 10.1167/iovs.08-2376 PMC2692837

[54] Li C-M., Presley J. B., Zhang X., Dashti N., Chung B. H., Medeiros N. E., Guidry C., and Curcio C. A. 2005 Retina expresses microsomal triglyceride transfer protein: implications for age-related maculopathy. J. Lipid Res. 46: 628–640. 15654125 10.1194/jlr.M400428-JLR200

[55] Penfold P. L., Killingsworth M., and Sarks S. H. 1985 Senile macular degeneration: the involvement of immunocompetent cells. Graefes Arch. Clin. Exp. Ophthalmol. 223: 69–76. 2408968 10.1007/BF02150948

[56] Sniderman A. D., Qi Y., Ma C-I. J., Wang R. H. L., Naples M., Baker C., Zhang J., Adeli K., and Kiss R. S. 2013 Hepatic cholesterol homeostasis is the low-density lipoprotein pathway a regulatory or a shunt pathway? Arterioscler. Thromb. Vasc. Biol. 33: 2481–2490. 23990208 10.1161/ATVBAHA.113.301517

[57] Zheng W., Reem R. E., Omarova S., Huang S., DiPatre P. L., Charvet C. D., Curcio C. A., and Pikuleva I. A. 2012 Spatial distribution of the pathways of cholesterol homeostasis in human retina. PLoS One. 7: e37926. 22629470 10.1371/journal.pone.0037926 PMC3358296

[58] Wang S., Xu L., Jonas J. B., Wang Y. X., You Q. S., and Yang H. 2012 Dyslipidemia and eye diseases in the adult Chinese population: the Beijing eye study. PLoS One. 7: e26871. 22128290 10.1371/journal.pone.0026871 PMC3419255

[59] Cougnard-Grégoire A., Delyfer M-N., Korobelnik J-F., Rougier M-B., Le Goff M., Dartigues J-F., Barberger-Gateau P., and Delcourt C. 2014 Elevated high-density lipoprotein cholesterol and age-related macular degeneration: the Alienor study. PLoS One. 9: e90973. 24608419 10.1371/journal.pone.0090973 PMC3946623

[60] Reynolds R., Rosner B., and Seddon J. M. 2010 Serum lipid biomarkers and hepatic lipase gene associations with age-related macular degeneration. Ophthalmology. 117: 1989–1995. 20888482 10.1016/j.ophtha.2010.07.009 PMC3081670

[61] van Leeuwen R., Vingerling J., Hofman A., De Jong P., and Stricker B. C. 2003 Cholesterol lowering drugs and risk of age related maculopathy: prospective cohort study with cumulative exposure measurement. BMJ. 326: 255–256. 12560276 10.1136/bmj.326.7383.255 PMC140763

[62] Chuo J. Y., Wiens M., Etminan M., and Maberley D. A. 2007 Use of lipid-lowering agents for the prevention of age-related macular degeneration: a meta-analysis of observational studies. Ophthalmic Epidemiol. 14: 367–374. 18161610 10.1080/09286580701421684

[63] Vavvas D. G., Daniels A. B., Kapsala Z. G., Goldfarb J. W., Ganotakis E., Loewenstein J. I., Young L. H., Gragoudas E. S., Eliott D., and Kim I. K. 2016 Regression of some high-risk features of age-related macular degeneration (AMD) in patients receiving intensive statin treatment. EBioMedicine. 5: 198–203. 27077128 10.1016/j.ebiom.2016.01.033 PMC4816836

[64] Bassen F. A., and Kornzweig A. L. 1950 Malformation of the erythrocytes in a case of atypical retinitis pigmentosa. Blood. 5: 381–387. 15411425

[65] Jampel R. S., and Falls H. F. 1958 Atypical retinitis pigmentosa, acanthrocytosis, and heredodegenerative neuromuscular disease. AMA Arch. Ophthalmol. 59: 818–820. 13532088 10.1001/archopht.1958.00940070032002

[66] Salt H. B., Wolff O. H., Lloyd J. K., Fosbrooke A. S., Cameron A. H., and Hubble D. V. 1960 On having no beta-lipoprotein. A syndrome comprising a-beta-lipoproteinaemia, acanthocytosis, and steatorrhoea. Lancet. 2: 325–329. 13745738 10.1016/s0140-6736(60)91478-1

[67] Brosnahan D. M., Kennedy S. M., Converse C. A., Lee W. R., and Hammer H. M. 1994 Pathology of hereditary retinal degeneration associated with hypobetalipoproteinemia. Ophthalmology. 101: 38–45. 8302562 10.1016/s0161-6420(94)31358-3

[68] Lin J. B., Mast N., Bederman I. R., Li Y., Brunengraber H., Björkhem I., and Pikuleva I. A. 2016 Cholesterol in mouse retina originates primarily from in situ de novo biosynthesis. J. Lipid Res. 57: 258–264. 26630912 10.1194/jlr.M064469 PMC4727421

[69] Saadane A., Mast N., Charvet C. D., Omarova S., Zheng W., Huang S. S., Kern T. S., Peachey N. S., and Pikuleva I. A. 2014 Retinal and nonocular abnormalities in Cyp27a1−/− Cyp46a1−/− mice with dysfunctional metabolism of cholesterol. Am. J. Pathol. 184: 2403–2419. 25065682 10.1016/j.ajpath.2014.05.024 PMC4188134

[70] Duncan K. G., Hosseini K., Bailey K. R., Yang H., Lowe R. J., Matthes M. T., Kane J. P., LaVail M. M., Schwartz D. M., and Duncan J. L. 2009 Expression of reverse cholesterol transport proteins ATP-binding cassette A1 (ABCA1) and scavenger receptor BI (SR-BI) in the retina and retinal pigment epithelium. Br. J. Ophthalmol. 93: 1116–1120. 19304587 10.1136/bjo.2008.144006 PMC3541028

[71] Lu J., Hübner K., Nanjee M. N., Brinton E. A., and Mazer N. A. 2014 An in-silico model of lipoprotein metabolism and kinetics for the evaluation of targets and biomarkers in the reverse cholesterol transport pathway. PLOS Comput. Biol. 10: e1003509. 24625468 10.1371/journal.pcbi.1003509 PMC3952822

[72] Paalvast Y., Kuivenhoven J. A., and Groen A. K. 2015 Evaluating computational models of cholesterol metabolism. Biochim. Biophys. Acta. 1851: 1360–1376. 26143380 10.1016/j.bbalip.2015.05.008

[73] Fritsche L. G., Igl W., Bailey J. N. C., Grassmann F., Sengupta S., Bragg-Gresham J. L., Burdon K. P., Hebbring S. J., Wen C., and Gorski M. 2016 A large genome-wide association study of age-related macular degeneration highlights contributions of rare and common variants. Nat. Genet. 48: 134–143. 26691988 10.1038/ng.3448 PMC4745342

[74] Cheng C-Y., Yamashiro K., Chen L. J., Ahn J., Huang L., Huang L., Cheung C. M. G., Miyake M., Cackett P. D., and Yeo I. Y. 2015 New loci and coding variants confer risk for age-related macular degeneration in East Asians. Nat. Commun. 6: 6063. 25629512 10.1038/ncomms7063 PMC4317498

[75] Pressly T. A., Scott W. J., Ide C. H., Winkler A., and Reams G. P. 1987 Ocular complications of Tangier disease. Am. J. Med. 83: 991–994. 3314502 10.1016/0002-9343(87)90667-x

[76] Hoffman H. N., and Fredrickson D. S. 1965 Tangier disease (familial high density lipoprotein deficiency): clinical and genetic features in two adults. Am. J. Med. 39: 582–593. 5831900 10.1016/0002-9343(65)90081-1

[77] Ng D. S., O’Connor P. W., Mortimer C. B., Leiter L. A., Connelly P. W., and Hegele R. A. 1996 Retinopathy and neuropathy associated with complete apolipoprotein AI deficiency. Am. J. Med. Sci. 312: 30–33. 8686727 10.1097/00000441-199607000-00006

