# Supplementary material for: An in silico model of retinal cholesterol dynamics (RCD model): insights into the pathophysiology of dry AMD
Source: J Lipid Res. 2017 Apr 25;58(7):1325–37. doi: 10.1194/jlr.M074088 (PMC5496031; doi:10.1194/jlr.M074088)
Supplement: Supplemental Data [file supp_58_7_1325__index.html]

An In-silico model of retinal cholesterol dynamics (RCD Model): Insights into the pathophysiology of dry age-related macular degeneration — An in silico model of retinal cholesterol dynamics (RCD model): insights into the pathophysiology of dry AMD — Supplemental Data 

# An in silico model of retinal cholesterol dynamics (RCD model): insights into the pathophysiology of dry AMD

## Supplemental Data

- Supplemental Material (.pdf, 595 KB) - Supplemental Material
