# Supplementary material for: An in silico model of retinal cholesterol dynamics (RCD model): insights into the pathophysiology of dry AMD
Source: J Lipid Res. 2017 Apr 25;58(7):1325–37. doi: 10.1194/jlr.M074088 (PMC5496031; doi:10.1194/jlr.M074088)
Supplement: Supplemental Data [file 10.1194_M074088_jlr.M074088-1.pdf]

Supplementary Material:

In-silico model of retinal cholesterol dynamics (RCD Model):
Insights into the pathophysiology of dry age-related macular degeneration

Seyedeh Maryam Zekavat[1, a]; James Lu[2, b]; Cyrille Maugeais[3]; and Norman A.Mazer[2, c]

1. Biological Engineering, Massachusetts Institute of Technology, Cambridge, MA, USA;
2. Clinical Pharmacology, Roche Innovation Center Basel, Basel, Switzerland;
3. Department of Neuroscience, Ophthalmology and Rare Diseases, Roche Innovation Center Basel, Basel, Switzerland

a) Current Address: Program in Medical and Population Genetics, Broad Institute, Cambridge, MA, USA; Center for Human Genetic Research and Cardiovascular Research Center, Massachusetts General Hospital, Boston, MA, USA

b) Current Address: Department of Drug Safety and Metabolism, AstraZeneca, Cambridge, UK

c) Author to whom correspondence should be addressed: norman.mazer@roche.com

Derivations and references on flux rates and other calculations made in the Retinal Cholesterol Dynamics (RCD) model (Sections 1 – 10).

Supplemental Material S1.Transit-chain model of Cholesterol (Ch) turnover in the Rod Outer Segment (ROS):

As depicted in Figure 2, we have used a transit-chain model (1) to describe the formation and movement of Ch-containing discs through the ROS and to compute the associated fluxes of Ch into and out of the discs. The input rate of Ch needed to maintain the Ch gradient in the ROS discs (K_{in}^{Ch}), when expressed per mm^2 of retina, defines the Ch turnover rate in the RCD model. Based on Young's study of the Rhesus monkey (2) we take the number of discs per ROS to be ~1000 and the rate at which discs enter the ROS (K_{in}^{discs}) to be ~85 discs/day. We divide the ROS into 10 compartments, each containing ~100 discs, which transit through the ROS and are phagocytosed by the RPE from the last compartment by a first-order process with rate constant k_t. The number of compartments has been chosen arbitrarily but has a negligible impact on the steady-state behavior of the system. The value of k_t is readily given by the steady-state condition ($K_{in}^{discs} = 100\ k_t$):

$$k_t = \frac{85\ discs/day}{100\ discs} = 0.85\ day^{-1} \qquad (S1.1)$$

It may be noted that the average time for a disc to transit from the first compartment to the RPE is equal to $\frac{10}{k_t}$ or 11.7 days as found by Young (2). The uncertainties of K_{in}^{discs} and k_t are estimated to be 6%.

With regard to Ch, Fig. 2 shows that the rate of Ch input to compartment *1* is derived from two sources. The first source, K_{in}^{Ch}, is the zero-order Ch flux delivered from the RPE to the RIS (including the possibility of Ch synthesis). The second source is the Ch flux delivered from the "recyclable pool", and is given by the product of the rate constant k_2 and the amount of Ch in the recyclable pool $C_{recyclable}^{Ch}$. The recyclable pool itself is supplied by Ch that is transferred out of the discs in compartments *2* through *10* with rate constant k_{out}. In addition to the Ch that is taken up by the RPE through the phagocytosis of discs in compartment *10*, we assume that Ch may also enter the RPE directly from the recyclable pool by a first-order process with rate constant k_1. The extent to which Ch is actually recycled from the recyclable pool to the RIS will depend on the ratio $\frac{k_2}{k_1+k_2}$, which we define as the recycling fraction $f_{recycling}$. In the following derivation we first show how k_{out} can be estimated from the concentration gradient of Ch in the ROS discs using the data from Boesze-Battaglia (3, 4). We then derive an expression for the dependence of K_{in}^{Ch} on $f_{recycling}$.

For compartment *1*, the steady-state condition is given by:

$$K_{in}^{Ch} + \ k_2 C_{recyclable}^{Ch} = k_t C_1^{Ch} \qquad (S1.2)$$

For transit-chain compartments *i=2-10* the steady-state condition is given by:

$$k_t C_{i-1}^{Ch} = (k_t + k_{out}) C_i^{Ch} \qquad (S1.3)$$

In these equations C_i^{Ch} is the total amount of Ch contained in the 100 discs of compartment *i*. Iterating Eq. *S*1.3 from compartment *1* to *i* leads to:

$$C_i^{Ch} = \left(\frac{k_t}{k_t + k_{out}}\right)^{i-1} C_1^{Ch} \qquad (S1.4)$$

For the recyclable pool, the steady-state condition is given by:

$$k_{out} \sum_{i=2}^{10} C_i^{Ch} = (k_1 + k_2) C_{recyclable}^{Ch} \qquad (S1.5)$$

which leads to:

$$C_{recyclable}^{Ch} = \frac{k_{out}}{k_1 + k_2} \sum_{i=2}^{10} C_i^{Ch} \qquad (S1.6)$$

Defining $\alpha = \frac{k_t}{k_t + k_{out}}$, and combining Eq. $S1.6$ with Eq. $S1.4$ we obtain:

$$C_{recyclable}^{Ch} = \frac{k_{out}}{k_1 + k_2} C_1^{Ch} (\alpha + \alpha^2 + \cdots + \alpha^9) \qquad (S1.7)$$

Summing the geometric series in $S1.7$ yields:

$$C_{recyclable}^{Ch} = \frac{k_{out}}{k_1 + k_2} C_1^{Ch} \frac{\alpha(1-\alpha^9)}{1-\alpha} \qquad (S1.8)$$

From Eq. $S1.4$, we may estimate α, from the experimentally derived ratio of Ch content in the "oldest" discs (compartment 10) vs. "newest" discs (compartment 1) of the ROS (3, 4):

$$\frac{C_{10}^{Ch}}{C_1^{Ch}} = \alpha^9 \qquad (S1.9)$$

As the ratio $\frac{C_{10}^{Ch}}{C_1^{Ch}}$ is approximately 1/6 with an estimated uncertainty of 55% (based on data in Boesze-Battaglia et al. (3)), the value of α is given by:

$$\alpha = \left(\frac{1}{6}\right)^{\frac{1}{9}} = 0.82 \qquad (S1.10)$$

with an estimated uncertainty of 6%. From this value and the previously estimated k_t we compute k_{out} to be 0.19 $day^{-1}$with an estimated uncertainty of 34%.

Combining Eq. $S1.2$ and Eq. $S1.8$, we arrive at the following expressions for K_{in}^{Ch}, the Ch turnover rate in the ROS:

$$K_{in}^{Ch} = k_t C_1^{Ch} - k_2 \frac{k_{out}}{k_1 + k_2} C_1^{Ch} \frac{\alpha(1-\alpha^9)}{1-\alpha} \qquad (S1.11)$$

Since $\frac{\alpha}{1-\alpha} = \frac{k_t}{k_{out}}$, this simplifies to:

$$K_{in}^{Ch} = k_t C_1^{Ch} \left[1 - \left(\frac{k_2}{k_1 + k_2}\right)(1-\alpha^9)\right] \qquad (S1.12)$$

Substituting $f_{recycling} = \frac{k_2}{k_1 + k_2}$ and combining with Eq. $S1.9$, we obtain the following result for K_{in}^{Ch}:

$$K_{in}^{Ch} = k_t C_1^{Ch} \left[1 - f_{recycling}\left(1 - \frac{C_{10}^{Ch}}{C_1^{Ch}}\right)\right] \qquad (S1.13)$$

As expected this equation shows that in the limit where $f_{recycling} = 0$ (no recycling), $K_{in}^{Ch} = k_t C_1^{Ch}$ while in the limit where $f_{recycling} = 1$ (complete recycling), $K_{in}^{Ch} = k_t C_{10}^{Ch}$.

Finally since $\frac{C_{10}^{Ch}}{C_1^{Ch}} \approx \frac{1}{6}$, Eq. $S1.13$ becomes:

$$K_{in}^{Ch} = k_t C_1^{Ch} \left[1 - \frac{5}{6} f_{recycling}\right] \qquad (S1.14)$$

This simple result shows that K_{in}^{Ch} will decrease linearly with $f_{recycling}$, from a maximum value of $k_t C_1^{Ch}$ to a minimum value that is 1/6 as large.

The numerical value of K_{in}^{Ch} requires an estimate of C_1^{Ch}, i.e., the amount of Ch contained in the 100 newly formed discs of compartment *1*, which is equal to 100 times the number of Ch molecules in one newly formed disc $C_1^{Ch/disc}$. Using this variable and the steady-state condition K_{in}^{discs} = 100 k_t (from Eq. *S*1.1), *S*1.14 is equivalent to:

$$K_{in}^{Ch} = K_{in}^{discs} C_1^{Ch/disc} \left[1 - \frac{5}{6} f_{recycling}\right] \qquad (S1.15)$$

To express K_{in}^{Ch} as a Ch flux in pg/mm^2/min of retina, we multiply the right hand side of S1.15 by the surface density of ROS in the retina σ_{retina}^{ROS}, the molecular weight of Ch M_{Ch} (386.65 g/mol), and divide by Avogadro's number N_A (6.02 x 10^{23}):

$$K_{in}^{Ch} = K_{in}^{discs} C_1^{Ch/disc} \left[1 - \frac{5}{6} f_{recycling}\right] \sigma_{retina}^{ROS} \frac{M_{Ch}}{N_A} \qquad (S1.16)$$

The value of $C_1^{Ch/disc}$ can be estimated from compositional data on ROS discs as the product of the molar ratio of Ch-to-PL ($f_{Ch:PL}$), the molar ratio of PL-to-Rhodopsin ($f_{PL:Rhod}$), the surface density of Rhodopsin in the ROS membrane (σ_{memb}^{Rhod}) and the area of bilayer membrane contained in a single ROS disc (A_{disc}):

$$C_1^{Ch/disc} = f_{Ch:PL}\, f_{PL:Rhod}\, \sigma_{memb}^{Rhod}\, A_{disc} \qquad (S1.17)$$

Table *S*1 summarizes the values of $f_{Ch:PL}$, $f_{PL:Rhod}$, σ_{memb}^{Rhod} and A_{disc} based on multiple sources from which we calculate $C_1^{Ch/disc}$ to be ~1.6 x 10^6 Ch molecules per disc (with an estimated uncertainty of 44%). Substituting this value into Eq. S1.16 (and dividing by 1440 minutes per day) we find that the numerical value of K_{in}^{Ch} is given by:

$$K_{in}^{Ch} = 5.84 \left[1 - \frac{5}{6} f_{recycling}\right] \approx 6 - 5\, f_{recycling} \qquad (pg/mm^2/min) \qquad (S1.18)$$

Thus K_{in}^{Ch} is ~6 pg/mm^2/min in the limit of no recycling ($f_{recycling} = 0$) and ~1 pg/mm^2/min in the limit of complete recycling ($f_{recycling} = 1$). Both values have an estimated uncertainty of 49%.

Table *S*1: Parameter values used in the Calculation of Retinal Cholesterol Turnover (Eq. *S*1.16 and *S*1.17)

Parameter	Definition	Units	Mean (uncertainty; %)[*]	Comments[†]
K_{in}^{discs}	The number of discs entering an ROS per day	discs/ROS/day	85 (6%)	From Rhesus monkey; mean and CV(%) from Table II (2).
σ_{retina}^{ROS}	Surface density of ROS in retinal tissue	ROS/mm^2	94,000 (20%)	From Rhesus monkey; mean and CV(%) of parafovea (73,000), perifovea (110,000), and peripheral areas (99,000) from Table III (2).
$C_1^{Ch/disc}$	Number of Ch molecules in a newly formed ROS disc	molecules/disc	1.64×10^6 (44%)	Calculated from the following parameters using Eq. *S*1.17; uncertainty calculated from CV(%) of each term (see footnote to this table).
$f_{Ch:PL}$	Molar ratio of cholesterol-to-phospholipid in newly formed ROS discs	mol/mol (dimensionless)	0.3 (17%)	From bovine retina (3, 4); uncertainty estimated visually from Figure 2B.
$f_{PL:Rhod}$	Molar ratio of phospholipid-to-rhodopsin in ROS disc	mol/mol (dimensionless)	72.3 (18%)	Mean and CV(%) of these values: 74; 54; 86; 75 (5-8).
σ_{memb}^{Rhod}	Surface density of rhodopsin in the ROS membrane	$molecules/\mu m^2$	29,800 (37%)	Mean and CV(%) of these values: 48,300; 25,000; 25,000; 30,000; 20,500. (9-13) Assumes a single bilayer area is $1.267\ \mu m^2$ (14).
A_{disc}	Membrane surface area of ROS disc (corresponds to 2 membranes per disc)	$\mu m^2/disc$	2.53 (0%)	Estimated as $2\pi\left(\frac{d_{ROS}}{2}\right)^2$; where d_{ROS} is the ROS diameter 1.27 µm (14).

[*] Uncertainty corresponds to the coefficient of variation, CV(%), for individual parameters. For composite parameters, e.g., $C_1^{Ch/disc}$, uncertainty was computed based on propagation of errors (15).
[†] Tables and figures mentioned refer to those within the referenced sources.

Supplemental Material S2. Transcytosis of LDL across the CC endothelium:

As discussed in section S6 (below), the effective pore size of the diaphragmed fenestra of the CC (16) ranges from ~6-12 nm, which would prevent the permeation of LDL particles, whose average diameter (17, 18) is ~21nm. We therefore assume that LDL passes across the CC endothelium by transcytosis (receptor-mediated vesicular transport) as suggested for other capillary beds (18-20). Based on Dehouk's in vitro study of the kinetics of LDL permeation across a brain endothelial monolayer (22), we derived the maximum flux rate (from the luminal to abluminal side) to be $21.7\,\frac{ng}{cm^2{*}hr}$. Dividing this by the LDL protein concentration on the donor (luminal) side of the monolayer, $50\,\frac{\mu g}{ml}$, we estimate the apparent permeability (23) of LDL across the CC endothelium P_{in}^{LDL} to be $\sim 1.2 \text{ x } 10^{-7}\,\frac{cm}{sec}$ (with an uncertainty of about 7%). Although Dehouk could not detect a comparable flux in the opposite direction (when donor solution was placed on the abluminal side of the monolayer), we assume that this permeability value (denoted P_{out}^{LDL}) can nevertheless vary from 0.1 to 1 times P_{in}^{LDL}. As shown in the next section and in Figure 6, the rate of receptor-mediated uptake of LDL by the RPE is relatively insensitive to the assumed value of P_{out}^{LDL}.

Supplemental Material S3. LDLR-mediated uptake into the RPE:

Based on experimental measurements (24, 25) and theoretical analysis (26) we model the receptor-mediated uptake of LDL particles by the RPE assuming Michaelis-Menten kinetics, given in Eq. S3.1:

$$V = \frac{(V_{max}^{LDLR} / A_{RPE}) \times C_{LDL}^{BrM}}{K_m^{LDLR} + C_{LDL}^{BrM}} \qquad (S3.1)$$

where V_{max}^{LDLR} is the maximum uptake rate per RPE cell, A_{RPE} is the basal surface area of an RPE cell, C_{LDL}^{BrM} is the LDL particle concentration in Bruch's membrane at the basal surface of the RPE, and K_m^{LDLR} is the LDL concentration corresponding to the half-maximal uptake rate. Defined in this way V is a flux rate that can be expressed in terms of LDL particle number, protein mass, or Ch mass using the fact that each LDL particle contains 1 ApoB molecule (MW = 500,000 g/mole) and ~2000 Ch molecules (27), taking the convention of expressing lipoprotein Ch mass as unesterified Ch (MW = 386.6 g/mole). We will begin expressing V using protein mass and convert to Ch mass at the end of the derivation.

Based on Hayes' *in vitro* study of LDL uptake in RPE monolayers (25) we estimate the value of K_m^{LDLR} to be ~35 μg/mL ApoB protein concentration (7 x 10^{-8} M), similar to the value of 8 x 10^{-8} M estimated in Hep-G2 cells by Harwood (26). Based on these two values the uncertainty in K_m^{LDLR} is estimated to be 9%. At a 10 μg/mL protein concentration in the incubation medium, Hayes found that "de-repressed" RPE cells (with maximal expression of LDL receptors) degraded 1200 ng protein/mg cell protein over 3 hrs, corresponding to an estimated degradation rate (that we equate with the *in vitro* V) of 6.67 ng protein/mg cell protein/min. The amount of cell protein in an RPE cell is estimated to be 9.5 x 10^{-7} mg based on a typical value for cellular protein concentration (25 g/mL) (28) and the RPE cell volume (3804 μm^3), calculated from A_{RPE} (336 μm^2) and the thickness (11.3 μm) of a parafoveal RPE cell in the rhesus monkey (2). Combining these values, the *in vitro* V corresponds to 6.34 x 10^{-3} pg protein/min per RPE cell. Dividing V by the ratio $\frac{C_{LDL}}{K_m^{LDLR}+C_{LDL}}$, or 0.22, we obtain V_{max}^{LDLR} for the de-repressed RPE cells to be 2.85 x 10^{-2} pg protein/min per RPE cell. Hayes further showed that the de-repressed state of the RPE monolayer could be down-regulated by 90% after a 24-hr incubation with LDL protein concentrations exceeding 20 μg/mL. Under physiological conditions in the BrM (where LDL protein is expected to be comparable to ApoB plasma concentrations, i.e., >500 μg/mL), we assume that V_{max}^{LDLR} will be down-

regulated 10-fold to ~2.85 x 10^{-3} pg protein/min per RPE cell. Dividing this value by A_{RPE} and converting to Ch mass we obtain our final estimate of V_{max}^{LDLR} / A_{RPE}, the maximal LDL-receptor mediated uptake rate, to be ~13.1 pg Ch/mm^2/min. Given the various assumptions made in this derivation, notably the degree of LDLR down regulation, we estimate the uncertainty in the value of V_{max}^{LDLR} / A_{RPE} to be 50%.

We now link Eq. *S3.1* with the transcytosis pathway discussed in Section 2, to model C_{LDL}^{BrM}, as in Eq. S3.2:

$$\frac{dC_{LDL}^{BrM}}{dt} = \left(P_{in}^{LDL} \times C_{LDL}^{CC} - P_{out}^{LDL} \times C_{LDL}^{BrM} - \frac{V_{max}^{LDLR}/A_{RPE} \times C_{LDL}^{BrM}}{K_m^{LDLR} + C_{LDL}^{BrM}}\right)\frac{1}{h_{BrM}} \qquad (S3.2)$$

where C_{LDL}^{CC} is the concentration of LDL in the choroidal capillaries and $h_{BrM}$is thickness of the BrM layer (approximated as 3 μm in the physiological state). As before we express LDL concentrations as mg Ch/dL and convert K_m^{LDLR} to 5.4 mg Ch/dL.

In the steady-state ($\frac{dC_{LDL}^{BrM}}{dt} = 0$), C_{LDL}^{CC} acts as a source term and C_{LDL}^{BrM} is determined by:

$$P_{in}^{LDL} \times C_{LDL}^{CC} = P_{out}^{LDL} \times C_{LDL}^{BrM} + \frac{V_{max}^{LDLR}/A_{RPE} \times C_{LDL}^{BrM}}{K_m^{LDLR} + C_{LDL}^{BrM}} \qquad (S3.3)$$

Solving Eq. S3.3 for C_{LDL}^{BrM} yields a quadratic equation which enables the LDL uptake rate into the RPE (the second term on the right side of Eq. $S3.3$) to be computed as a function of C_{LDL}^{CC}. This is shown graphically in Figure 6 for three values of the transcytosis permeability ratio $P_{out}^{LDL} / P_{in}^{LDL}$. All three curves approach the limit given by V_{max}^{LDLR}/A_{RPE} as C_{LDL}^{CC} exceeds 50 mg/dL.

Supplemental Material S4. ABCA1-mediated transport from apical RPE to outer retina

We estimate the rate of ABCA1-mediated transport of Ch from the apical surface of RPE cells to the outer retina, K_{in}^{ABCA1}, from the rate of hepatic ABCA1-mediated transport via the Reverse Cholesterol Transport (RCT) pathway, $K_{RCT}^{hepatocyte}$. The latter is estimated (per kg body weight, BW) by multiplying the whole-body rate of ABCA1-medated input to plasma HDL (32.9 mg Ch secreted to HDL/kg BW/day) (29) with the fraction of whole-body RCT provided by the liver (f_{Liver}^{RCT} = 0.734) (30), to obtain 24.1 mg Ch/kg BW/day. As the ratio of liver weight-to-total body weight is ~0.03 in mammals (31, 32), this corresponds to a $K_{RCT}^{hepatocyte}$ of 803 mg Ch/kg liver/day. To express this on a per hepatocyte basis we use the fact that hepatocytes comprise ~80% of liver weight and represent the hepatocyte as a cube with a side length (31, 32) of ~19.3 μm and a density of 1 gm/cm^3. The resulting value of $K_{RCT}^{hepatocyte}$ is 7.22 x 10^{-9} mg Ch/hepatocyte/day. We convert this to a flux rate across the basal and apical surfaces of the hepatocyte (mg Ch/mm^2/min) by dividing it by twice the area of the cube face, i.e., 2 x (19.3 μm)^2, and converting from days to minutes to obtain 6.73 pg Ch/min/mm^2. We take this value to be our estimate of K_{in}^{ABCA1} by assuming that the RPE has a comparable density of ABCA1 transporters on its apical surface as the hepatocyte has on its apical and basal surfaces. Given the various assumptions in this derivation, notably the last one, we estimate the uncertainty of K_{in}^{ABCA1} to be 50%.

Supplemental Material S5. ABCA1-mediated efflux from basal RPE to BrM:

The rate of ABCA1-mediated Ch efflux from the basal surface of the RPE, K_{out}^{ABCA1}, was estimated as being a fraction of the previously estimated flux through the apical surface, K_{in}^{ABCA1}, using Eq. ($S5.1$):

$$K_{out}^{ABCA1} \approx \frac{1}{f_{Apical/Basal}^{ABCA1\ expression}} K_{in}^{ABCA1} \approx \frac{1}{6.37} K_{in}^{ABCA1} \qquad (S5.1)$$

The fraction (1/6.37) was determined from the relative expression of ABCA1 in the apical vs. basal RPE of the mouse retina from Ananth et al. (33) using ImageJ. As depicted in Fig. S5.1, the ratio of the integrated areas under the image fluorescence intensity curves from the apical-to-basal sides of the RPE was determined in 12 cross-sections. The mean ratio is shown in the corresponding box plot.

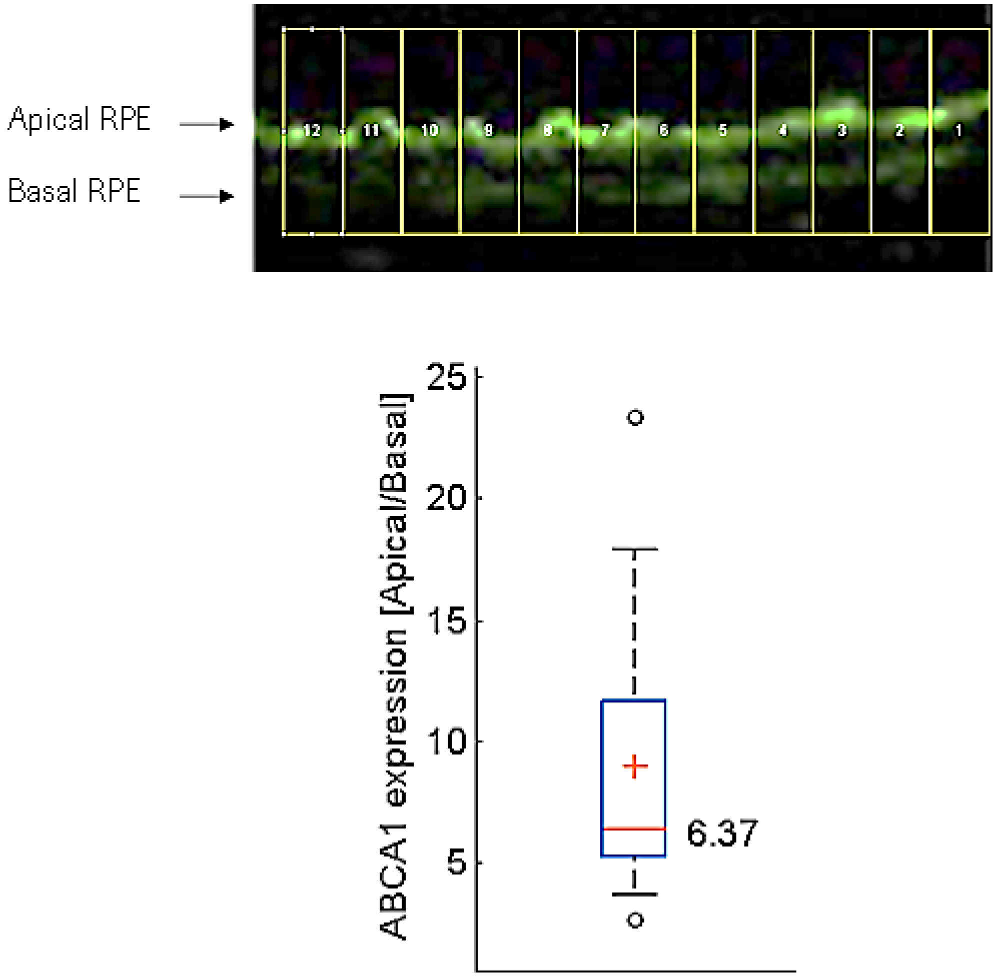

Figure S5.1: Fractional expression of ABCA1 on apical vs. basal RPE. Upper panel shows ABCA1 staining in RPE from Ananth et al. (33) and segmentation in ImageJ. In the lower panel, the box plot shows the distribution of apical-to-basal expression ratios across all segments with a mean of 6.37.

The resulting estimated value of K_{out}^{ABCA1} is 1.06 pg Ch/ mm^2/min. As in section S5, we estimate the uncertainty of K_{out}^{ABCA1} to be 50%.

S6. Effective permeability of ApoA-I-Ch from BrM to CC via diaphragmed endothelial fenestra

We first show that the ApoA-I-Ch particles effluxed via the ABCA1 transporter from the RPE or Drusen-associated macrophages are small enough to pass through the pores of the diaphragmed fenestrations of

the CC endothelium, whose maximum size (16) has been estimated to be ~12 nm (uncertainty 4%). The ApoA-I-Ch particles, which are analogous to nascent HDL discs or so called pre-beta particles, contain 2 ApoA-I molecules, ~20 unesterified Ch molecules and ~40 phospholipid molecules (29, 34). From this composition, the molecular weights and partial specific volumes of the constituent molecules, we have calculated the total volume of the particle, and, assuming a spherical shape, estimate its diameter to be ~6.3 nm (uncertainty 5%).

The permeability coefficient of the ApoA-I-Ch particles across the CC ($P_{ApoA1-Ch}^{CC}$) can be calculated using the theory for transport of particles through cylindrical pores (35-38).

$$P_{ApoA1-Ch}^{CC} = \left(\frac{\gamma\, D}{L}\right) H(\lambda) \qquad [S6.1]$$

$$\text{where: } H(\lambda) = \frac{6\pi(1-\lambda)^2}{K_t\,(\lambda)};\, if\ 0 \le \lambda < 1 \qquad [S6.2]$$

$$K_t(\lambda) = \frac{9}{4}\pi^2\sqrt{2} * (1-\lambda)^{-\frac{5}{2}}\,(1 + a_1(1-\lambda) + a_2(1-\lambda)^2) + a_3 + a_4\lambda + a_5\lambda^2 + a_6\lambda^3 + a_7\lambda^4 \qquad [S6.3]$$

$$a_1 = -\frac{73}{60};\, a_2 = \frac{77293}{50400};\ a_3 = -22.5083;\ a_4 = -5.6117;\ a_5 = -0.3363;\ a_6 = -1.216;\ a_7 = 1.647 \qquad [S6.4]$$

In these equations, γ represents the fraction of the vessel wall surface area occupied by pores, D represents the aqueous diffusion coefficient of the particles (calculated using the Einstein-Stokes relation), L represents the thickness of the vessel wall, and λ is the ratio of the particle size to pore size. $H(\lambda)$ represents the hydrodynamic interactions between the solute and pores (which attenuate the diffusivity within the pore) and $K_t(\lambda)$ is a function which uses the numerical values of the coefficients a_1 to a_7 given in Eq. S6.4. We estimate these parameters (and their uncertainties) as follows.

- γ = 39.1 fenestrations/μm^2 of endothelium[39] × 8 pores/ fenestration[40,41] × π (12 nm/2)^2 = 0.0354 (uncertainty 15%)
- D (37 °C, 0.15 M NaCl) = 1.02 x 10^{-6} cm^2/sec (using the Stokes-Einstein relation for a 6.3 nm diameter particle and the viscosity of physiological saline at 37 °C) (uncertainty 5%)
- L = 0.1 μm, the thickness of an endothelial cell (42) (uncertainty 20%)
- $\lambda = \frac{6.3\ nm\ diam}{12\ nm\ diam} = 0.525$ (uncertainty 6%)

The function $H(\lambda)$ is computed from Eq. $S6.2$, $S6.3$ and $S6.4$.

Using these parameter values and equations, Fig. S6.1 depicts how the permeability of the particle across the CC depends on λ.

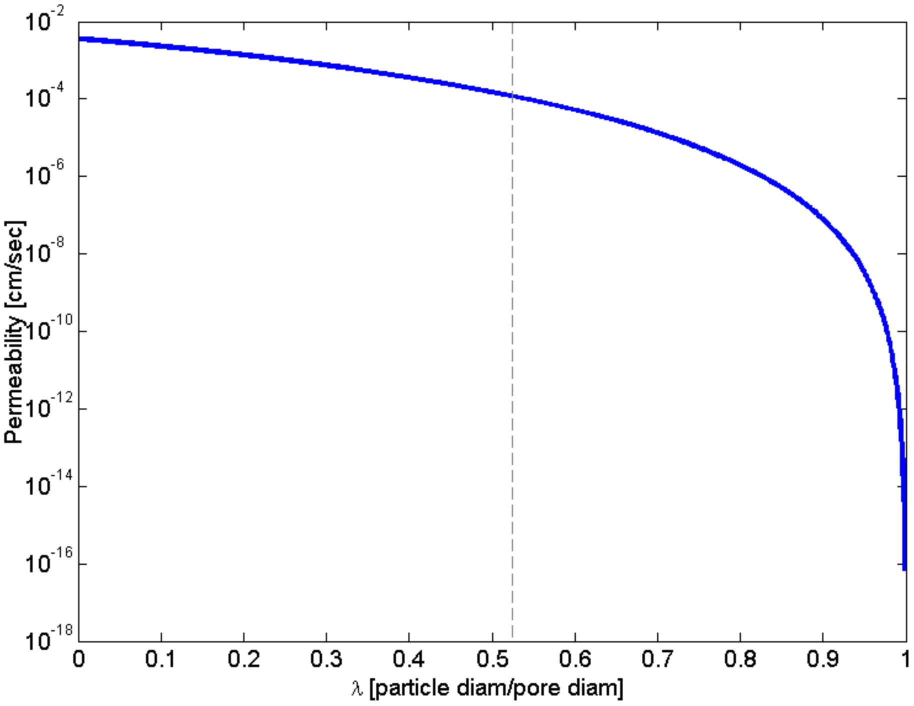

Fig. S6.1: Permeability of a particle passing through the diaphragmed fenestrations of the choroidal capillary as a function of the ratio of the particle diameter to pore diameter. Dashed verticle line corresponds to the ApoA-I-Ch particle (λ = 0.53).

For the 6.3 nm ApoA-I-Ch particle, the value of $P_{ApoA1-Ch}^{CC}$ is calculated to be $1.2 \times 10^{-4}\ cm/sec$. Based on the uncertainty in λ, the corresponding uncertainty in H(λ) and the uncertainties in γ, D and L, we estimate the overall uncertainty in $P_{ApoA1-Ch}^{CC}$ to be 41%. For particles approaching the pore size (12 nm) the permeability falls off sharply.

Assuming that ApoA-I-Ch particles enter the BrM either from the RPE or via ABCA1-efflux from Drusen-associated macrophages and that influx from the choroid capillary is negligible, e.g., sink conditions, we can estimate their concentration $\left(C_{ApoA1-Ch}^{BrM}\right)$ as follows:

$$\frac{dC_{ApoA1-Ch}^{BrM}}{dt} = (K_{ApoA1-Ch}^{RPE} + K_{ApoA1-Ch}^{Mac} - P_{ApoA1-Ch}^{CC} \times C_{ApoA1-Ch}^{BrM})\frac{1}{h_{BrM}} \qquad [S6.5]$$

where $K_{ApoA1-Ch}^{RPE}$ is the RPE derived influx rate of ApoA-I-Ch particles to the BrM and $K_{ApoA1-Ch}^{Mac}$ is the macrophage derived influx rate, both expressed in terms of the Ch mass associated with the ApoA-I-Ch particles (pg Ch/min/mm^2).

At steady-state $\left(\frac{dC_{ApoA1-Ch}^{BrM}}{dt} = 0\right)$, $C_{ApoA1-Ch}^{BrM}$ is given by:

$$C_{ApoA1-Ch}^{BrM} = \frac{(K_{ApoA1-Ch}^{RPE} + K_{ApoA1-Ch}^{Mac})}{P_{ApoA1-Ch}^{CC}} \qquad [S6.6]$$

Considering first that the RPE is only the source of ApoA-I-Ch particles and that $K_{ApoA1-Ch}^{RPE}$ corresponds to our previous estimate of K_{out}^{ABCA1}, i.e., ~1 pg/mm^2/min, the estimated value of $C_{ApoA1-Ch}^{BrM}$ is 0.0014 mg Ch/dL. Based on the composition of the ApoA-I-Ch particle given at the beginning of this section, this value corresponds to ~0.01 mg ApoA-I/dL. If $K_{ApoA1-Ch}^{RPE}$ were 6-fold larger (as in the non-recycling case, see section S1), $C_{ApoA1-Ch}^{BrM}$ would equal 0.0084 mg Ch/dL or ~0.06 mg ApoA-I/dL. These values are appreciably smaller than the concentration of lipid-poor ApoA-I in plasma (29), ~5mg ApoA-I/dL.

In the case where the macrophage efflux is the dominant source of the ApoA-I-Ch particles, our calculations (see Section S9 below) show that $K_{ApoA1-Ch}^{Mac}$ values as large as ~200 pg/mm^2/min may occur. The corresponding value of $C_{ApoA1-Ch}^{BrM}$ would equal 0.278 mg Ch/dL or 2.05 mg ApoA-I/dL.

Supplemental Material S7. ApoB-Ch Secretion out of the RPE into BrM

The efflux rate of ApoB-Ch particles from RPE to the BrM, $K_{out}^{ApoB-Ch}$ is estimated here based on the rate of hepatic ApoB efflux per hepatocyte ($K_{ApoB}^{hepatocyte}$) using an analogous approach to the ABCA1-mediated transport calculation in section S4. $K_{ApoB}^{hepatocyte}$ is derived from the rate of hepatic ApoB input to plasma (K_{ApoB}^{Liver}), measured in healthy subjects to be 24.8 mg ApoB/kg body weight/day (43). Using the same liver weight-to-body weight ratio as before (31, 32), 0.03, this corresponds to 827 mg ApoB/kg liver/day. Taking the molecular weight of ApoB to be 500,000 g/mole, we convert this to 1.65 x 10^{-6} moles ApoB/kg liver/day. Making the same geometric assumptions about hepatocyte as before, $K_{ApoB}^{hepatocyte}$ becomes 1.49 x 10^{-17} moles ApoB/hepatocyte/day or 1.99 x 10^{-14} moles ApoB/mm^2/day.

We now convert this flux rate to Ch based on the ~70 nm particle described by Curcio et al. (44). Taking the volume of the hydrophobic core of the particle to be the spherical volume of the particle and dividing by the molecular volume of a cholesteryl ester molecule (CE) (1.08 nm^3 (27)), we calculate that each particle will contain approximately 168,000 CE molecules. Based on Shen's 1977 model of spherical lipoprotein structure (27) we estimate that the 70 nm particles would have about 12,500 molecules of unesterified Ch molecules on the particle surface, yielding a total of 180,500 Ch molecules per particle. The corresponding Ch flux rate of ApoB-Ch particles (converted to the mass of unesterified Ch and expressed per minute), i.e., our estimate of $K_{out}^{ApoB-Ch}$, is 967 pg/mm^2/min.

In comparison to the previous calculations of K_{in}^{Ch} (see section S1), the estimate of $K_{out}^{ApoB-Ch}$ is approximately two orders of magnitude larger; indicating that if the RPE functioned like a hepatocyte it would be more than able to eliminate all of the Ch taken up daily from the ROS in the form of ApoB particles. However, according to Fujihara's study of transgenic mice with a human genomic fragment encoding full-length human ApoB (45), the RPE expression of ApoB is about 7.5% of the levels expressed in the liver. Similarly the RPE expression of the microsomal transport protein (MTP-A) mRNA, a key protein involved in ApoB secretion, is 4% of the levels expressed in the liver. Both levels appear to be regulated by the amount of intracellular CE, which is lower in the RPE compared to HepG2 cells (46). If these expression factors were to act in a multiplicative manner, it would imply that the synthesis and secretion of ApoB in the RPE would be reduced by a factor of 0.003. Acknowledging that mRNA expression levels may not directly correspond to protein levels, reducing the previous value by this factor would lead to a hypothesized $K_{out}^{ApoB-Ch}$ for RPE cells of ~3 pg/mm^2/min, which is comparable to the earlier estimate of turnover rate, K_{in}^{Ch}. Given the various assumptions involved in the derivation of $K_{out}^{ApoB-Ch}$, we estimate its uncertainty to be 50%.

Supplemental Material S8. Ch deposition rate in the BrM (Drusen growth rate):

The calculation of the Ch deposition rate in the BrM is based on a number of key assumptions. First, that the Ch deposited in the BrM originates from the Ch turnover of the ROS, i.e. K_{in}^{Ch}. Second, that this material is secreted from the RPE in 70 nm ApoB-containing particles with a core of CE and surface that contains unesterified Ch, i.e., $K_{out}^{ApoB-Ch} = K_{in}^{Ch}$. To be consistent with Curcio's analysis of the lipid composition of BrM deposits, we assume that about half of the secreted CE becomes unesterified (47). And third, that due to its 70 nm size the secreted particles cannot enter the CC and therefore become entrapped in the BrM as basal linear deposits and drusen, which are combined in our estimate of drusen growth rate. These assumptions imply that the amount of Ch deposited in the BrM will increase linearly with time and can therefore be characterized by a linear deposition rate $\frac{dh_{drusen}^{growth}}{dt}$ (expressed in µm per year) that is proportional to K_{in}^{Ch} (equivalently $K_{out}^{ApoB-Ch}$):

$$\frac{dh_{drusen}^{growth}}{dt} = 10^{-12} \times K_{in}^{Ch} \times \left[\frac{1}{2}\left(\frac{1}{\rho_C} + \frac{M_{CE}/M_C}{\rho_{CE}}\right)\right] \times 10^6 \times 525{,}600 = 0.709\, K_{in}^{Ch} \qquad [S8.1]$$

where ρ_C is the density of unesterified Ch (1 g/cm^3), ρ_{CE} is the density of esterified Ch (0.991 g/cm^3) and M_{CE} is the molecular weight of cholesterol ester (taken as 650 g/mol). The factor 10^{-12} converts the turnover rate from pg/mm^2/min to g/mm^2/min of retinal tissue; the term in brackets (corresponding to 1.35 cm^3/g) converts the amount of Ch in grams of unesterified Ch to an equivalent volume (in cm^3) of an equimolar mixture of UC and CE; the factor 10^6 converts the cm^3/mm^2 to µm; and the factor 525,600 converts the growth rate from µm/min to µm/year.

For values of K_{in}^{Ch} corresponding to the previously estimated range of 1 to 6 pg/mm^2/min, the corresponding range of $\frac{dh_{drusen}^{growth}}{dt}$ is 0.7 to 4.2 µm/year. In principal, the drusen growth rate could vary locally over the retina in proportion to the value of K_{in}^{Ch} in that region, e.g., at the high end of the range in the vicinity of large drusen and at the low end of the range in the vicinity of the basal linear deposits. If such deposition rates persisted for 5 decades, the resulting thickness of Ch deposited in the BrM would range from 35 to 210 µm. The uncertainty in the growth rates and thicknesses are estimated to be the same as for K_{in}^{Ch}, i.e., 49%.

Supplemental Material S9. Macrophage-mediated Ch clearance via the ABCA1 transport mechanism

In principle the rate-limiting step in macrophage-mediated clearance of drusen Ch could involve the phagocytosis of drusen Ch by macrophages, the movement of macrophages into or out of the BrM or the ABCA1-mediated efflux of Ch from macrophages to lipid-poor ApoA-I. An in-vitro study of the phagocytosis of polysterene spheres by macrophages (48) indicates that this rate is at least 5-fold faster than the ABCA1-medicated efflux rate of Ch (see later calculation). We have not found any data on the rates at which macrophages enter and/or leave the BrM nor is it known whether macrophages leave the BrM after phagocytosing the drusen material. We therefore assume that ABCA1-mediated Ch efflux is the rate-limiting step for drusen Ch clearance and model the efflux of Ch (v_{ABCA1}^{Mac}) from the BrM region to lipid-poor ApoA-I via the following Michaelis-Menten equation:

$$v_{ABCA1}^{Mac} = v_{max}^{Mac}\left(\frac{C_{BrM}^{ApoA-I}}{K_m^{Mac} + C_{BrM}^{ApoA-I}}\right) \qquad [S9.1]$$

where v_{max}^{Mac} and K_m^{Mac} are derived from in vitro Ch efflux studies of acetylated LDL-loaded macrophages to lipid poor ApoA-I (49-51) and C_{BrM}^{ApoA-I} is the lipid-poor ApoA-I concentration in the BrM. From these studies, K_m^{Mac} was found to be ~5 μg/mL (uncertainty 35%) and v_{max}^{Mac} was estimated to be 46.4 ng Ch/mg cell protein/min (uncertainty 15%).

We convert v_{max}^{Mac} to a per macrophage basis (pg Ch/cell/min) by estimating the mass of cell protein per macrophage using estimates of the of protein concentration in cells (0.25 g/cm^3) (28), and the volume per human macrophage (30) (4990 μm^3) and obtain 5.79 x 10^{-2} pg Ch/cell/min. It is interesting to compare this value to an estimate of the rate of phagocytosis of drusen cholesterol by macrophages, which we have derived from the rate of ingestion of polystyrene particles by activated macrophages *in-vitro* (48). In a 90-minute incubation, macrophages ingested a total volume of polystyrene microparticles of 118 μm^3/cell, corresponding to an estimated rate of ingestion equal to 1.31 pg/cell/min. Assuming that drusen mass is consumed at the same rate and contains 22% Ch (52), the calculated rate of Ch ingestion is 0.294 pg Ch/cell/min or 5.1 times greater than the previous estimate of the ABCA1-mediated Ch efflux rate.

Using this value, we calculate the rate of drusen clearance (in μm/year) as a function of the macrophage density in the BrM (C_{retina}^{Mac}) and lipid poor ApoA-I concentration by applying the same multiplicative factor of 0.709 from Eq. S8.1, thereby arriving at Eq *S*9.2.

$$\frac{dh_{Drusen}^{Mac}}{dt} = -\, v_{max}^{Mac}\, C_{retina}^{Mac} \left(\frac{C_{BrM}^{ApoA-I}}{K_m^{Mac} + C_{BrM}^{ApoA-I}} \right) 0.709 \qquad [S9.2]$$

In this context, the retinal density of macrophages refers to the number of macrophages per mm^2 that are located in the sub-RPE space in the vicinity of Drusen deposits and above the CC. The exact thickness of this region is somewhat arbitrary, but is taken in our calculations as 100 μm based on the tabulations of leukocyte concentrations in different strata of the human retina by Penfold et al. (53). Combining two of these strata corresponds to the 100 μm thickness.

As shown in Table S9.1 the leukocyte densities in the 100 μm thick region ranges from 149 to 1421 cells/mm^2 for six groups of subjects with increasing severity of retinal pathology. We note that the theoretically maximum density of macrophages corresponding to a hexagonal packing of spheres (with a volume of 4990 μm^3) is about 15,000 cells per mm^2 in a 100 μm region based on the maximum volume fraction of 0.74 from Kepler's Conjecture (54). Thus the experimental densities in Table S9.1 range from 1-10 % of this maximal macrophage density.

Table S9.1: Experimental leukocyte densities in sub-RPE strata and strata (ss and s) as reported by Penfold et al. (53) for 6 groups of patients with progressive AMD. Raw cell counts have been converted to cell/mm^2 based on the size of the areas counted. Total count corresponds to the 100 μm thickness between RPE and CC.

**Strata (distance from CC into BrM):**	**Group I: Normal Aging**	**Group II: Progressive AMD (early)**	**Group III: Progressive AMD (intermediate)**	**Group IV: Progressive AMD (late)**	**Group V: Geographic Atrophy**	**Group VI: Disciform degeneration**
ss (0-10 μm)	17	142	217	217	546	892
s (10-100 μm)	132	467	483	434	378	529
ss + s (0-100 μm)	149	608	699	651	923	1421

Based on this data and the theoretical upper limit we consider that the macrophage density could conceivably range from 0 to 5000 cells/mm^2 in the 100 μm thickness of the BrM containing a large drusen.

We have used Eq. *S*9.2 to simulate the rate of drusen clearance (decrease in height vs. time) for different values of macrophage density (C_{retina}^{Mac}) in the above range and various fold-changes in the macrophage ABCA1 activity (v_{max}^{Mac}) and the concentration of lipid-poor ApoA-I in the BrM (C_{BrM}^{ApoA-I}) (see Results: Macrophage-mediated clearance of drusen). The baseline values of the 3 parameters were: C_{retina}^{Mac} = 1000 cells/mm^2; v_{max}^{Mac} = 5.79 x 10^{-2} pg/cell/min; and C_{BrM}^{ApoA-I} = 25 μg/mL. The uncertainty in $\frac{dh_{Drusen}^{Mac}}{dt}$ is assumed to be the same as v_{max}^{Mac} (27%).

In order to compare these simulations with serial OCT data on the rapid clearance of a drusen in a patient with dry AMD (55) the initial height of the drusen was taken to be 120 μm.

Supplemental Material S10. Analysis of Lin's Study of Retinal Ch Metabolism in the Mouse

In a recently published study, Lin et al. have experimentally determined the Ch content of the mouse retina to be 1.13 μg per g of wet tissue and the Ch input rate to be 21 μg per g of wet tissue per day (56). We re-normalize these values from g of wet tissue to mm^2 of retina by assuming a tissue density of 1 g/cm^3 and taking the total retinal thickness to be 200 μm based on Ferguson et al (57). The re-normalized values correspond to a Ch content of 226 ng/mm^2 retina and a Ch input rate of 2.9 pg/mm^2/min. We now compare these experimental values to estimates from the RCD model with adjustment for the higher rod density in the mouse retina (500,000 ROS/mm^2) and smaller number of discs per ROS (600 discs/ROS) (58). The corresponding values in the rhesus monkey are a rod density of 94,000 ROS/mm^2 and 1000 discs/ROS (see section S1).

Assuming the average Ch/PL ratio of the discs across the length of the ROS to be 0.12 (see section S1) (3), we estimate the Ch content in the mouse outer retina to be 126 ng/mm^2 retina. This corresponds to 56% of the experimental value, which seems reasonable as the latter value would also include the contribution from the neural cell layers of the inner retina.

To estimate the Ch turnover in the mouse we further adjust the disc turnover rate to be 60 per day (vs. 85 per day in the rhesus monkey), based on the 10-day transit time measured in mouse (58). Using the RCD

model we obtain a lower limit for Ch turnover in the mouse of 3.7 pg/mm^2/min (corresponding to complete recycling) and an upper limit of 21.9 pg/mm^2/min (corresponding to no recycling). As the previously estimated uncertainty in the Ch turnover calculations is 49%, we consider the estimated range and experimental Ch input rate (2.9 pg/mm^2/min) to be in reasonable agreement. Although Lin et al. find that Ch synthesis is the major source of Ch input in the mouse (which could be a species-specific finding), our analysis of Ch content and turnover nevertheless shows that their data are consistent with the structural and dynamic assumptions of the RCD model.

References:

1. Savic, R. M., D. M. Jonker, T. Kerbusch, and M. O. Karlsson. 2007. Implementation of a transit compartment model for describing drug absorption in pharmacokinetic studies. *J. Pharmacokinet. Pharmacodyn.* **34**: 711-726.
2. Young, R. W. 1971. The renewal of rod and cone outer segments in the rhesus monkey. The *Journal of Cell Biology* **49**: 303-318.
3. Boesze-Battaglia, K., T. Hennessey, and A. D. Albert. 1989. Cholesterol heterogeneity in bovine rod outer segment disk membranes. *J. Biol. Chem.* **264**: 8151-8155.
4. Boesze-Battaglia, K., S. J. Fliesler, and A. D. Albert. 1990. Relationship of cholesterol content to spatial distribution and age of disc membranes in retinal rod outer segments. *J. Biol. Chem.* **265**: 18867-18870.
5. Daemen, F. J. 1973. Vertebrate rod outer segment membranes. *Biochimica et Biophysica Acta (BBA)-Reviews on Biomembranes* **300**: 255-288.
6. Stone, W., C. Farnsworth, and E. Dratz. 1979. A reinvestigation of the fatty acid content of bovine, rat and frog retinal rod outer segments. *Exp. Eye Res.* **28**: 387-397.
7. Calvert, P., V. Govardovskii, N. Krasnoperova, R. Anderson, J. Lem, and C. Makino. 2001. Membrane protein diffusion sets the speed of rod phototransduction. *Nature* **411**: 90-94.
8. Miljanich, G., P. Nemes, D. White, and E. Dratz. 1981. The asymmetric transmembrane distribution of phosphatidylethanolamine, phosphatidylserine, and fatty acids of the bovine retinal rod outer segment disk membrane. *The Journal of membrane biology* 60: 249-255.
9. Fotiadis, D., Y. Liang, S. Filipek, D. A. Saperstein, A. Engel, and K. Palczewski. 2003. Atomic-force microscopy: rhodopsin dimers in native disc membranes. *Nature* **421**: 127-128.
10. Pugh, E., and T. Lamb. 1993. Amplification and kinetics of the activation steps in phototransduction. *Biochimica et Biophysica Acta (BBA)-Bioenergetics* **1141**: 111-149.

11. Liebman, P. A., K. R. Parker, and E. A. Dratz. 1987. The molecular mechanism of visual excitation and its relation to the structure and composition of the rod outer segment. *Annu. Rev. Physiol.* **49**: 765-791.

12. Roof, D. J., and J. E. Heuser. 1982. Surfaces of rod photoreceptor disk membranes: integral membrane components. *The Journal of cell biology* **95**: 487-500.

13. Buzhynskyy, N., C. Salesse, and S. Scheuring. 2011. Rhodopsin is spatially heterogeneously distributed in rod outer segment disk membranes. *J. Mol. Recognit.* **24**: 483-489.

14. Nickell, S., P. S.-H. Park, W. Baumeister, and K. Palczewski. 2007. Three-dimensional architecture of murine rod outer segments determined by cryoelectron tomography. *The Journal of cell biology* **177**: 917-925.

15. Palmer M. Experimental Analysis: Propagation of Uncertainty through Mathematical Operations. 2003. [updated 2003 January 21; accessed 2016 April 18]. http://web.mit.edu/fluids-modules/www/exper_techniques/2.Propagation_of_Uncertaint.pdf

16. Sarin, H. 2010. Physiologic upper limits of pore size of different blood capillary types and another perspective on the dual pore theory of microvascular permeability. *J. Angiogenes. Res.* **2**: 1.

17. Jeyarajah, E. J., W. C. Cromwell, and J. D. Otvos. 2006. Lipoprotein particle analysis by nuclear magnetic resonance spectroscopy. *Clin. Lab. Med.* **26**: 847-870.

18. Mora, S., J. D. Otvos, N. Rifai, R. S. Rosenson, J. E. Buring, and P. M. Ridker. 2009. Lipoprotein particle profiles by nuclear magnetic resonance compared with standard lipids and apolipoproteins in predicting incident cardiovascular disease in women. *Circulation* **119**: 931-939.

19. Brown, M. S., and J. L. Goldstein. 1979. Receptor-mediated endocytosis: insights from the lipoprotein receptor system. *Proceedings of the National Academy of Sciences* **76**: 3330-3337.

20. Frank, P. G., S. Pavlides, and M. P. Lisanti. 2009. Caveolae and transcytosis in endothelial cells: role in atherosclerosis. *Cell Tissue Res.* **335**: 41-47.

21. von Eckardstein, A., and L. Rohrer. 2009. Transendothelial lipoprotein transport and regulation of endothelial permeability and integrity by lipoproteins. *Curr. Opin. Lipidol.* **20**: 197-205.

22. Dehouck, B., L. Fenart, M.-P. Dehouck, A. Pierce, G. Torpier, and R. Cecchelli. 1997. A new function for the LDL receptor: transcytosis of LDL across the blood–brain barrier. *The Journal of cell biology* **138**: 877-889.

23. Brodin, B., B. Steffansen, and C. U. Nielsen. 2010. Passive diffusion of drug substances: the concepts of flux and permeability. *In* Molecular biopharmaceutics. B. Steffansen, B.Brodin and C.U. Nielsen, Editors. Pharmaceutical Press, London. 135-151.

24. Brown, M. S., and J. L. Goldstein. 1975. Regulation of the activity of the low density lipoprotein receptor in human fibroblasts. *Cell* **6**: 307-316.

25. Hayes, K., S. Lindsey, Z. Stephan, and D. Brecker. 1989. Retinal pigment epithelium possesses both LDL and scavenger receptor activity. Invest. *Ophthalmol. Vis. Sci.* **30**: 225-232.

26. Harwood, H. J., and L. D. Pellarin. 1997. Kinetics of low-density lipoprotein receptor activity in Hep-G2 cells: derivation and validation of a Briggs–Haldane-based kinetic model for evaluating receptor-mediated endocytotic processes in which receptors recycle. *Biochem. J.* **323**: 649-659.

27. Shen, B.-W., A. Scanu, and F. Kezdy. 1977. Structure of human serum lipoproteins inferred from compositional analysis. *Proceedings of the National Academy of Sciences* **74**: 837-841.

28. Milo, R. 2013. What is the total number of protein molecules per cell volume? A call to rethink some published values. *Bioessays* **35**: 1050-1055.

29. Lu, J., K. Hübner, M. N. Nanjee, E. A. Brinton, and N. A. Mazer. 2014. An in-silico model of lipoprotein metabolism and kinetics for the evaluation of targets and biomarkers in the reverse cholesterol transport pathway. *PLoS Comput. Biol.* **10**: e1003509.

30. van de Pas, N. C., R. A. Woutersen, B. van Ommen, I. M. Rietjens, and A. A. de Graaf. 2012. A physiologically based in silico kinetic model predicting plasma cholesterol concentrations in humans. *J. Lipid Res.* **53**: 2734-2746.

31. Prothero, J. W. 1982. Organ scaling in mammals: the liver. *Comparative Biochemistry and Physiology Part A: Physiology* **71**: 567-577.

32. Sohlenius-Sternbeck, A.-K. 2006. Determination of the hepatocellularity number for human, dog, rabbit, rat and mouse livers from protein concentration measurements. *Toxicol. In Vitro* **20**: 1582-1586.

33. Ananth, S., J. P. Gnana-Prakasam, Y. D. Bhutia, R. Veeranan-Karmegam, P. M. Martin, S. B. Smith, and V. Ganapathy. 2014. Regulation of the cholesterol efflux transporters ABCA1 and ABCG1 in retina in hemochromatosis and by the endogenous siderophore 2, 5-dihydroxybenzoic acid. *Biochimica et Biophysica Acta (BBA)-Molecular Basis of Disease* **1842**: 603-612.

34. Duong, P. T., G. L. Weibel, S. Lund-Katz, G. H. Rothblat, and M. C. Phillips. 2008. Characterization and properties of preβ-HDL particles formed by ABCA1-mediated cellular lipid efflux to apoA-I. *J. Lipid Res.* **49**: 1006-1014.

35. Bungay, P. M., and H. Brenner. 1973. The motion of a closely-fitting sphere in a fluid-filled tube. *International Journal of Multiphase Flow* **1**: 25-56.

36. Deen, W. M., C. R. Bridges, B. M. Brenner, and B. D. Myers. 1985. Heteroporous model of glomerular size selectivity: application to normal and nephrotic humans. *American Journal of Physiology-Renal Physiology* **249**: F374-F389.

37. Deen, W. 1987. Hindered transport of large molecules in liquid-filled pores. *AIChE J.* **33**: 1409-1425.

38. Stylianopoulos, T., K. Soteriou, D. Fukumura, and R. K. Jain. 2013. Cationic nanoparticles have superior transvascular flux into solid tumors: insights from a mathematical model. *Ann. Biomed. Eng.* **41**: 68-77.

39. Ishibashi, R., A. Sugita, and H. Yoshioka. 1984. Dynamic changes of fenestrations in choriocapillaries. *The Kurume medical journal* **31**: 309-315.

40. Melamed, S., I. Ben-Sira, and Y. Ben-Shaul. 1980. Ultrastructure of fenestrations in endothelial choriocapillaries of the rabbit--a freeze-fracturing study. *The British journal of ophthalmology* **64**: 537.

41. Johnson, M., A. Dabholkar, J.-D. Huang, J. B. Presley, M. F. Chimento, and C. A. Curcio. 2007. Comparison of morphology of human macular and peripheral Bruch's membrane in older eyes. *Curr. Eye Res.* **32**: 791-799.

42. Aird, W. C. 2007. Phenotypic heterogeneity of the endothelium I. Structure, function, and mechanisms. *Circ. Res.* **100**: 158-173.

43. Maugeais, C., K. Ouguerram, M. Krempf, P. Maugeais, J. Gardette, E. Bigot, and T. Magot. 1996. A minimal model using stable isotopes to study the metabolism of apolipoprotein B-containing lipoproteins in humans. *Diabetes Metab.* **22**: 57-63.

44. Wang, L., C.-M. Li, M. Rudolf, O. V. Belyaeva, B. H. Chung, J. D. Messinger, N. Y. Kedishvili, and C. A. Curcio. 2009. Lipoprotein particles of intraocular origin in human Bruch membrane: an unusual lipid profile. *Invest. Ophthalmol. Vis. Sci.* **50**: 870-877.

45. Fujihara, M., E. Bartels, L. B. Nielsen, and J. T. Handa. 2009. A human apoB100 transgenic mouse expresses human apoB100 in the RPE and develops features of early AMD. *Exp. Eye Res.* **88**: 1115-1123.

46. Li, C.-M., J. B. Presley, X. Zhang, N. Dashti, B. H. Chung, N. E. Medeiros, C. Guidry, and C. A. Curcio. 2005. Retina expresses microsomal triglyceride transfer protein: implications for age-related maculopathy. *J. Lipid Res.* **46**: 628-640.

47. Curcio, C. A., M. Johnson, J.-D. Huang, and M. Rudolf. 2010. Apolipoprotein B-containing lipoproteins in retinal aging and age-related macular degeneration. *J. Lipid Res.* **51**: 451-467.

48. Pacheco, P., D. White, and T. Sulchek. 2013. Effects of microparticle size and Fc density on macrophage phagocytosis. *PLoS One* **8**: e60989.

49. Kritharides, L., W. Jessup, E. L. Mander, and R. T. Dean. 1995. Apolipoprotein AI–mediated efflux of sterols from oxidized LDL–loaded macrophages. *Arterioscler. Thromb. Vasc. Biol.* **15**: 276-289.

50. Liu, L., A. E. Bortnick, M. Nickel, P. Dhanasekaran, P. V. Subbaiah, S. Lund-Katz, G. H. Rothblat, and M. C. Phillips. 2003. Effects of Apolipoprotein AI on ATP-binding cassette transporter A1-mediated efflux of macrophage phospholipid and cholesterol formation of nascent high density lipoprotein particles. *J. Biol. Chem.* **278**: 42976-42984.

51. Sankaranarayanan, S., G. Kellner-Weibel, M. DE La Llera-Moya, M. C. Phillips, B. F. Asztalos, R. Bittman, and G. H. Rothblat. 2011. A sensitive assay for ABCA1-mediated cholesterol efflux using BODIPY-cholesterol. *J. Lipid Res.* **52**: 2332-2340.

52. Wang, L., M. E. Clark, D. K. Crossman, K. Kojima, J. D. Messinger, J. A. Mobley, and C. A. Curcio. 2010. Abundant lipid and protein components of drusen. *PLoS One* **5**: e10329.

53. Penfold, P., M. Killingsworth, and S. Sarks. 1985. Senile macular degeneration: the involvement of immunocompetent cells. *Graefe's archive for clinical and experimental ophthalmology* **223**: 69-76.

54. Hales, T. C. 1992. The sphere packing problem. *Journal of Computational and Applied Mathematics* **44**: 41-76.

55. Ouyang, Y., F. M. Heussen, A. Hariri, P. A. Keane, and S. R. Sadda. 2013. Optical coherence tomography–based observation of the natural history of drusenoid lesion in eyes with dry age-related macular degeneration. *Ophthalmology* **120**: 2656-2665.

56. Lin, J. B., N. Mast, I. R. Bederman, Y. Li, H. Brunengraber, I. Björkhem, and I. A. Pikuleva. 2016. Cholesterol in mouse retina originates primarily from in situ de novo biosynthesis. *J. Lipid Res.* **57**: 258-264.

57. Ferguson, L. R., J. M. Dominguez II, S. Balaiya, S. Grover, and K. V. Chalam. 2013. Retinal thickness normative data in wild-type mice using customized miniature SD-OCT. *PLoS One* **8**: e67265.

58. Volland, S., L. C. Hughes, C. Kong, B. L. Burgess, K. A. Linberg, G. Luna, Z. H. Zhou, S. K. Fisher, and D. S. Williams. 2015. Three-dimensional organization of nascent rod outer segment disk membranes. Proceedings of the National Academy of Sciences 112: 14870-14875.
